# The relationship between extreme inter-individual variation in macrophage gene expression and genetic susceptibility to inflammatory bowel disease

**DOI:** 10.1007/s00439-024-02642-9

**Published:** 2024-02-29

**Authors:** Claire L. O’Brien, Kim M. Summers, Natalia M. Martin, Dylan Carter-Cusack, Yuanhao Yang, Rasel Barua, Ojas V. A. Dixit, David A. Hume, Paul Pavli

**Affiliations:** 1 Centre for Research in Therapeutics Solutions, Faculty of Science and Technology, University of Canberra, Canberra, ACT Australia; 2 Inflammatory Bowel Disease Research Group, Canberra Hospital, Canberra, ACT Australia; 3 Mater Research Institute-University of Queensland, Translational Research Institute, Brisbane, QLD Australia; 4 School of Medicine and Psychology, College of Health and Medicine, Australian National University, Canberra, ACT Australia

## Abstract

The differentiation of resident intestinal macrophages from blood monocytes depends upon signals from the macrophage colony-stimulating factor receptor (CSF1R). Analysis of genome-wide association studies (GWAS) indicates that dysregulation of macrophage differentiation and response to microorganisms contributes to susceptibility to chronic inflammatory bowel disease (IBD). Here, we analyzed transcriptomic variation in monocyte-derived macrophages (MDM) from affected and unaffected sib pairs/trios from 22 IBD families and 6 healthy controls. Transcriptional network analysis of the data revealed no overall or inter-sib distinction between affected and unaffected individuals in basal gene expression or the temporal response to lipopolysaccharide (LPS). However, the basal or LPS-inducible expression of individual genes varied independently by as much as 100-fold between subjects. Extreme independent variation in the expression of pairs of HLA-associated transcripts (*HLA-B/C, HLA-A/F* and *HLA-DRB1/DRB5*) in macrophages was associated with HLA genotype. Correlation analysis indicated the downstream impacts of variation in the immediate early response to LPS. For example, variation in early expression of *IL1B* was significantly associated with local SNV genotype and with subsequent peak expression of target genes including *IL23A, CXCL1, CXCL3, CXCL8* and *NLRP3*. Similarly, variation in early *IFNB1* expression was correlated with subsequent expression of IFN target genes. Our results support the view that gene-specific dysregulation in macrophage adaptation to the intestinal milieu is associated with genetic susceptibility to IBD.

**Supplementary Information:**

The online version contains supplementary material available at 10.1007/s00439-024-02642-9.

## Introduction

Susceptibility to the major chronic inflammatory bowel diseases (IBD), Crohn’s disease (CD) and ulcerative colitis, is heritable. The strongest associations are with the human major histocompatibility gene complex (HLA), contributing up to 30% of shared risk amongst siblings for CD and even greater for ulcerative colitis (Ahmad et al. 2006; Ashton et al. 2019). In terms of mechanism, there is strong evidence that IBD arises from hyper-responsiveness of cells of the monocyte–macrophage lineage to gut microbiota (Graham and Xavier 2020; Jostins et al. 2012; Sudhakar et al. 2021). Rare monogenic early-onset forms of IBD are commonly due to mutations in monocyte–macrophage-expressed genes (Liu and Stappenbeck 2016; Loddo and Romano 2015; Nambu et al. 2022; Uhlig et al. 2014). Modern population genome-wide association studies (GWAS) have identified hundreds of additional non-HLA variants associated with significantly increased risk of developing IBD (Huang et al. 2017; Jostins et al. 2012; Lee et al. 2017; Liu et al. 2015). Collectively, this genetic variation still explains < 20% of the total population disease variance (Torres et al. 2017). Polygenic risk scores based upon GWAS data have limited clinical predictive value; even amongst those with the highest relative genetic risk, the absolute lifetime risk is low (Gettler et al. 2021). Siblings presumably share some components of both genotype and environment. However, concordance amongst dizygotic twins (who may share a more similar environment compared to siblings) may be < 5% and even for monozygotic twins shared risk is estimated at only 15–30% (Cho and Brant 2011; Halme et al. 2006). The difference between estimated heritability based upon family studies and relative risk based upon genotype analysis has been attributed in part to limitations in sample size and other constraints on GWAS and to complex interactions between genes and environment. To extend association analysis, whole genome sequencing has been applied to identify rare and/or causal variants within expression quantitative trait loci (eQTL) (Momozawa et al. 2018; Sazonovs et al. 2022; Somineni et al. 2021). After sequencing 100 candidate genes in 6600 CD patients and 5500 controls, Momozawa et al. (2018) concluded that > tenfold greater sample sizes would be required to demonstrate causality. Accordingly, Sazonovs et al. (2022) analyzed whole exome or whole genome sequences of > 30,000 CD patients and 80,000 controls to implicate coding variation in an additional ten genes in susceptibility.

There have been numerous hypothesis-driven candidate gene analyses providing evidence of associations between IBD and single nucleotide variants (SNVs) associated with inflammatory mediators. For example, Richard et al. (2018) identified a disease-associated functional regulatory variant linked to reduced expression of *TNFSF15* (TL1A) which has been implicated in gut inflammation. Recently, Klunk et al. (2022) proposed that the medieval plague epidemic led to selection for variants affecting immune response gene expression. They highlighted a SNV linked to expression of the *ERAP2* gene in macrophages*.* The same SNV is reported to be associated with IBD risk (Di Narzo et al. 2016).

Identification of disease-relevant transcriptional regulation requires analysis of a disease-associated cell population. The intestinal lamina propria contains an abundant population of resident tissue macrophages which turn over constantly and are replaced by recruitment of blood monocytes. Following their entry to the mucosal niche, monocytes must rapidly modulate their response to the products of microorganisms to avoid inflammatory activation. The progressive adaptation of monocytes to the intestinal environment has been described as a waterfall (Hegarty et al. 2023; Joeris et al. 2017; Mowat et al. 2017). Bsat et al. (2020) described a system to replicate this cascade based upon sequential cultivation in CSF2 (GM-CSF), interferon-gamma and IL23. However, this system reflects an inflammatory milieu. The differentiation of resident lamina propria macrophages in mouse and human depends upon macrophage colony-stimulating factor (CSF1) (Hegarty et al. 2023) and these cells are rapidly depleted by administration of anti-CSF1R antibody (MacDonald et al. 2010). Accordingly, the differentiation of human monocytes in vitro in response to CSF1 and their subsequent response to an archetypal microbial agonist such as lipopolysaccharide (LPS) may provide a more informative model of differentiation of monocyte-derived resident macrophages in the ileum and colon (Baillie et al. 2017). Based upon extensive gene expression analysis in monocytes and macrophages produced through the FANTOM5 consortium (Forrest et al. 2014), loci associated with IBD were shown to be strongly and specifically enriched for promoters that were regulated during CSF1-driven monocyte differentiation or activation by LPS (Baillie et al. 2017, 2018). This conclusion was supported by subsequent studies integrating GWAS signals with monocyte gene expression data (Gettler et al. 2019).

In this study, we used the CSF1/LPS treatment model for the adaptation of monocytes to the intestinal milieu to test the hypothesis that there are consistent and identifiable genetically determined differences in monocyte–macrophage regulatory responses that distinguish affected from unaffected individuals. Based upon inducible expression of an NF-κB-regulated luciferase in patient macrophages Papoutsopoulou et al. (2019) claimed that CD patients were hyper-responsive to TLR4 signalling. In this study we have found no evidence to support this conclusion. Instead, we demonstrate that monocyte-derived macrophages (MDM) from both affected and unaffected individuals within families affected by IBD display extreme variation in constitutive or inducible expression of individual genes and classes of genes, notably including the HLA complex. We suggest that such extreme variation could predispose to dysregulation of the response to microbial challenge and hence affect risk of developing IBD in response to an environmental trigger.

## Materials and methods

All patients provided consent and the study was approved by the ACT Health Human Research Ethics Committee (ETH.5.07.464).

### Blood monocyte isolation

Approximately 65 mL of blood was drawn from each subject into ACD-B Vacuette® tubes, transferred to sterile 50 mL tubes, and centrifuged for 30 min at 12,000 × g with no brake. A 200 μL aliquot of blood was kept for DNA extraction. Plasma was removed from the tubes so that only a 1 cm layer remained. Approximately 9 mL of buffy coat was aspirated from each sample and diluted 1/1.5 in RPMI with no additives. The buffy coat dilution was gently layered on to Lymphoprep™ to a total volume of 50 mL in a 50 mL tube, then spun for 45 min at 200 × g at room temperature with no brake. The clear top layer was removed and the mononuclear cells (peripheral blood mononuclear cells; PBMC) were recovered, diluted in 30 mL RPMI1640 and pelleted by centrifugation. Cells were counted, washed once with phosphate buffered saline. CD14-positive cells were enriched by using the Classical Monocyte Isolation Kit (Miltenyi 130-117-337) according to the manufacturer’s instructions. A 10 μL aliquot of the magnetic column eluate was removed for cell counts and purity check. The remainder of the elution was centrifuged for 10 min at 300 × g at 4 °C.

### Cell culture

The CD14^+^ monocyte pellet was resuspended in complete culture medium; RPMI1640 (Sigma-Aldrich) containing 2 mM Glutamax (Gibco), 20 ug/ml penicillin/streptomycin (Sigma-Aldrich), and 10% v/v human AB serum (Sigma-Aldrich). Recombinant human macrophage colony-stimulating factor (CSF1, Gibco-Thermo Fisher, PHC9501) was then added at a final concentration of 100 ng/mL. The cells were then plated at 10^6^ cells per well, to a final volume of 2 mL. The 6-well plates had been coated with 1 μg/cm^2^ human plasma fibronectin (Sigma-Aldrich) as per the manufacturer’s instructions to create a more physiologically relevant substratum. The 6-well plates were placed in the incubator at 37 °C with 5% CO_2_. At day four of incubation 1 mL of fresh complete medium with 3 × CSF1 concentration was added to each well. On day six, the medium was replaced with 2 mL complete medium with 1× concentration of CSF1. On day seven of incubation, the time course experiments commenced (Day 1), 10 ng/ml lipopolysaccharide (LPS) *Salmonella enterica* serotype Minnesota Re 595 (Sigma-Aldrich) was added to each well. Time point 0 h was collected before LPS was added. At each time point (2 h, 7 h, 21 h), cells were lysed using 0.5 mL TRIzol (Invitrogen), after which 100 μL chloroform was added. The plates were rocked back and forth for 15 s and incubated at 37 °C with 5% CO_2_ for 2 min. The cell lysates were then placed in sterile microcentrifuge tubes and placed at − 80 °C.

### Transcriptome sequencing

A total of 224 samples underwent whole genome transcriptome sequencing. Lysed cell samples were thawed and RNA extracted using a Qiagen RNeasy miniprep kit, including DNase digestion (Qiagen). RNA concentration and purity was assessed on a Tapestation (Agilent). Library preparation was performed using Illumina TruSeq Stranded mRNA kits. Sequencing was performed on a NovaSeq 6000 instrument using S1 flowcells (three in total) in a 100 bp paired-end format. Library preparation and sequencing were performed by the Biomolecular Resource Facility of the Australian National University, Canberra, Australia, according to manufacturer’s instructions.

### SNV genotyping

DNA was extracted from a 200 μL blood sample for each subject using QIAamp DNA Blood Mini kits with RNaseA digestion (Qiagen). SNV genotyping was performed using Illumina Infinium ImmunoArray-24v2.0 BeadChips (Illumina, Inc., San Diego, CA, USA). A total of 4 μL DNA was loaded onto the array, as per the manufacturer’s guidelines. The BeadChips were scanned using an Illumina iScan. Samples were genotyped using Illumina’s GenomeStudio 2.0.4 with Genotyping module 2.0.4 software, using default settings. The Illumina InfiniumImmunoArray-24v2-0 manifest file and project specific cluster file were used for the analysis (InfiniumImmunoArray-24v2-0_A_ClusterFile.egt). SNV genotyping was performed by Australian Genome Research Facility, Melbourne, Australia.

### Bioinformatics

FastQC (https://www.bioinformatics.babraham.ac.uk/projects/fastqc/) was used to assess the quality of RNA-seq results. Assignment of reads to transcripts was then conducted using the pseudoaligner Kallisto v0.44 (Bray et al. 2016). Ensembl release 103 of the GRCh38 genome was used for all kmer matching and subsequent annotation. The R package tximeta (Love et al. 2020) was used to calculate gene-level expression, corrected for effective gene length, resulting in a file for abundance, in counts per million (CPM). Ensembl gene and primary transcript IDs, NCBI stable gene IDs, Ensembl gene symbols and HGNC symbols were included for each gene. This preparation of the data for analysis was carried out by the ANU Bioinformatics Consultancy, Australian National University, Canberra, Australia. We then verified assignment of the RNA-seq time point samples to individuals using RNA2HLA (Chelysheva et al. 2021) which also allowed HLA-A, HLA-B, HLA-C and HLA-DRB1 typing. Assignment of sex was validated using expression of Y chromosome genes.

Network analysis was then performed on the abundance file. In the final data set there were 222 records from 56 individuals; two samples (one from the 2 h time point of an affected individual and one from the 21 h time point of an unaffected sibling) failed QC and were removed from the analysis. Lowly expressed genes (CPM < 1.0) and genes expressed in < 2 sample records were removed. The resultant dataset consisted of 19,972 genes. The network analysis tool *BioLayout* (http://biolayout.org, formerly BioLayout *Express*^3D^ (Theocharidis et al. 2009) was used to visualize the transcriptomic data and explore relationships between samples (sample-to-sample analysis) and genes (gene-to-gene analysis). For the sample-to-sample analysis, a threshold Pearson correlation coefficient of 0.87 was used; this was the maximum value that included all samples. For gene-to-gene analysis, a Pearson correlation coefficient of 0.70 was chosen to maximise the number of genes included whilst minimizing the computational complexity due to the number of connections between them (Bush et al. 2018). Similar results were obtained when the data were clustered using the non-parametric Spearman correlation coefficient (*r* > 0.89 for sample-to-sample analysis and *r* > 0.70 for gene-to-gene analysis).

Analysis of the SNV genotypes was performed by Australian Genome Research Facility, Melbourne, Australia. The GRCh38 build of the human reference genome was used for analysis. Final reports were merged and converted to PLINK PED/MAP format using gcta v1.91.2. Alleles were flipped to the positive strand using the ImmunoArray v2.0 strand designations. Quality control was carried out using PLINK (Purcell et al. 2007). Filtered SNVs were pruned by pairwise genetic correlation to create a set of independent variants appropriate for principal component analysis (PCA). LD pruning was calculated using a window size of 1000 bp and 50 bp step with r^2^ threshold of 0.5. Annotated PCA plots were generated using the ggplot2 package (Ginestet-C 2011) in the R statistical environment.

Expression QTL (eQTL) analysis was performed for 56 individuals at 4 time points using Matrix-eQTL version 2.3 (Shabalin 2012) in the R statistical environment version 4.1.2. The testing window for identification of *cis*-acting variants was 10^6^ bp. The expression levels were taken from the same file used for network analysis. The four timepoints were modelled in linear mode (least squares). A stringent minor allele frequency threshold of 0.2 was applied to reduce inflation generated by rare alleles (Wang et al. 2021). Sites with a significance score of *p* < 1 × 10^–5^ were exported. Quantile–quantile plots and *p* value histograms were generated to assess the effectiveness of the model and data QC.

Polygenic risk score (PRS) analysis was performed as described (Choi et al. 2020) using the ImmunoArray genotypes. IBD-specific SNPs for PRS calculation were selected according to the IBD GWAS summary statistics (Liu et al. 2015) at different GWAS *p* value thresholds (at 5 × 10^–8^, 1 × 10^–6^, 1 × 10^−4^, 1 × 10^–3^, 0.01, 0.05, 0.1, 0.3, 0.5 and 1). Independent SNPs were then filtered by linkage disequilibrium (LD) clumping with LD *r*^2^ < 0.05 within window size at 1000 Kb. PRS was calculated through PLINK ‘score’ function (Purcell et al. 2007) and subsequently normalised.

### Statistical analysis

Associations between genotype and gene expression were analyzed using non-parametric tests because of skew in the data. The Kruskal–Wallis test was used for three genotypes and the Mann–Whitney *U* test for two genotypes. Associations between expression of two genes were assessed using the Spearman correlation coefficient. Analysis was performed using GraphPad Prism v.9.

## Results

### Description of the cohort and polygenic risk score

Blood monocyte gene expression is clearly impacted by disease status and treatment (Gettler et al. 2019). The extended cultivation of isolated monocytes in CSF1 in vitro to generate MDM is intended to mitigate those impacts to enable detection of genotype-related variation. MDM were prepared from a total of 56 individuals, including affected and unaffected sib pairs/trios from 22 IBD families and a set of six healthy individuals with no known family history of IBD, including one sib-pair (Table S1A). All were genotyped using the Illumina Infinium ImmunoArray BeadChip v2.0. This microarray comprises 196,524 SNPs and small indel markers selected on the basis of GWAS results from 12 different immune-mediated diseases including IBD, supplemented with additional SNPs as described (Liu et al. 2015). All of the individuals in this cohort were identified as being of European ethnicity. Consistent with that identity, PCA using all SNPs (at a minor allele frequency of 0.01) separated only 3 sets of siblings (Fig. S1A). A single sib trio (MN27) separated in the first component and two additional families (MN23 and MN21) in the second component, whereas all other individuals were tightly grouped. We used the genotype data to calculate normalized polygenic risk scores (PRS) for IBD based upon published IBD GWAS summary statistics (Liu et al. 2015). The results for individual subjects are shown alongside the complete patient metadata in Table S1A. Of the 11 unaffected individuals with a positive PRS, 9 had an affected sibling who had a positive PRS of similar magnitude. Figure S1B shows the distribution of calculated PRS for affected and unaffected individuals and the decline in PRS with less stringent GWAS *p* value thresholds. As expected, given the known contribution of HLA to disease risk, the average PRS was reduced when HLA-associated SNPs were excluded (Fig. S1C). There was no significant difference in average PRS between affected and unaffected individuals, nor in pairwise comparison of affected individuals and their unaffected siblings, likely because of the small cohort and relatedness. However, the distribution of calculated PRS is consistent with published data for individuals of European descent (Gettler et al. 2021).

### Network analysis of the response of monocyte-derived macrophages to LPS

The response of human MDM to LPS is a sequential cascade of transient induction and repression of sets of transcripts (Baillie et al. 2017). Based upon the previous dense time course analysis using Cap Analysis of Gene Expression (CAGE) tag sequencing (Baillie et al. 2017), MDM populations were sampled at zero time, and 2, 7 and 21 h after addition of a maximally effective concentration (100 ng/mL) of *Salmonella Minnesota* Re595 LPS, a pure TLR4 agonist (Bush et al. 2020; Schroder et al. 2012). The chosen time points represent the peaks of expression of early, mid and late response genes, also applied in previous comparative analysis of LPS response in mouse and human (Schroder et al. 2012) and in multiple other mammalian species (Bush et al. 2020). RNA-seq was performed on mRNA extracted at each time point and initially quantified using Kallisto as described previously (Bush et al. 2020). The complete dataset organized by individual and family is provided in Table S1B.

To overview the data, we initially performed a network analysis using *BioLayout* (http://biolayout.org) (Theocharidis et al. 2009). A further development of this platform *Graphia* (https://graphia.app/) was published recently (Freeman et al. 2022). By contrast to the widely-used weighted gene correlation analysis (WGCNA) approach (Langfelder and Horvath 2008) this network method generates an all vs. all correlation matrix to which it applies a correlation coefficient (*r*) threshold cutoff removing outliers. The thresholded matrix is used to generate a true correlation graph from which coexpression modules are then defined using the Markov clustering (MCL) algorithm, which further refines and removes outliers. The outcomes are similar to WGCNA, but modules defined by this approach are biologically enriched over those defined by WGCNA (Freeman et al. 2022).

### Sample-to-sample network does not distinguish between affected and unaffected individuals

**Fig. 1:**
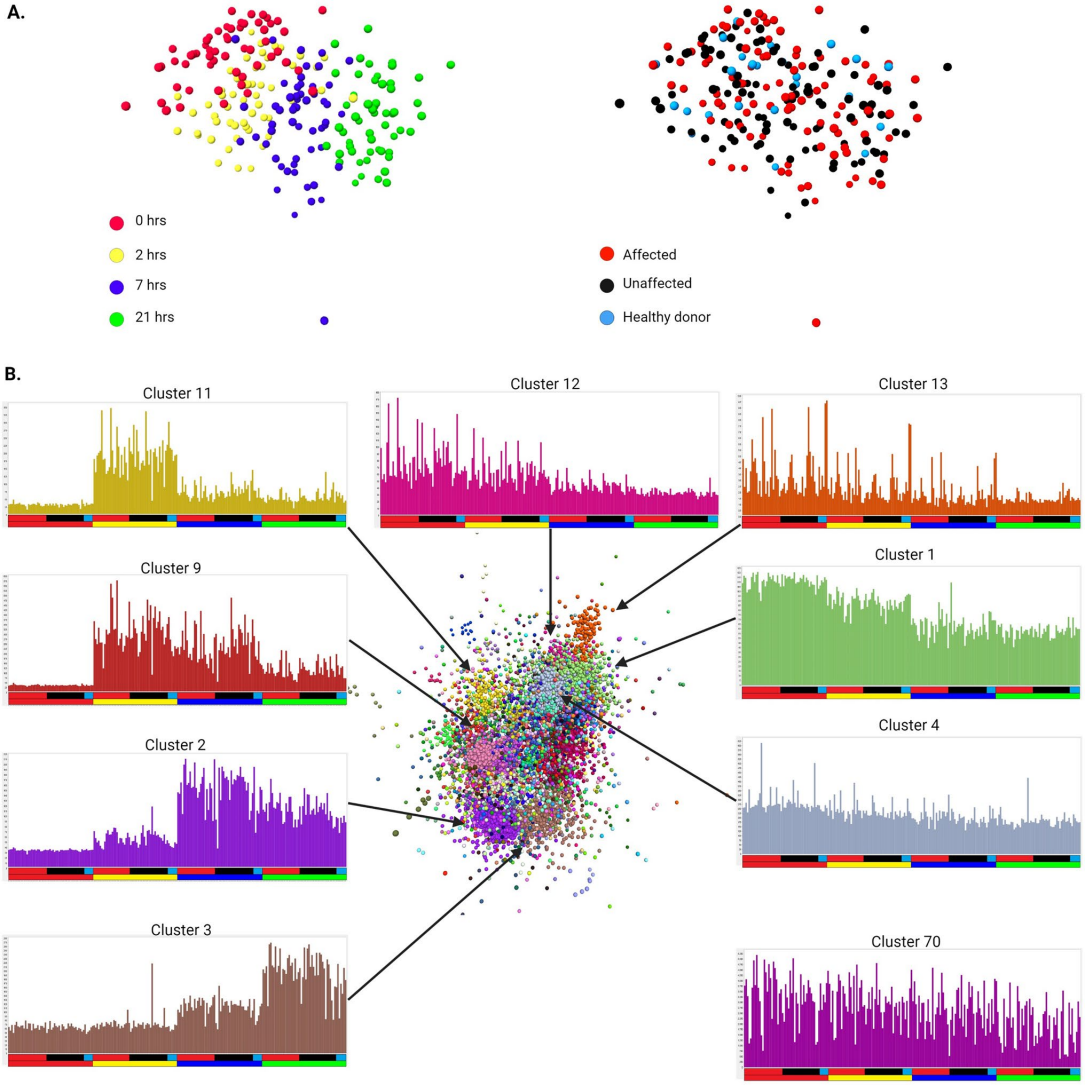
Network analysis of gene expression in macrophages stimulated with LPS. Gene expression profiles from RNA-seq analysis of the time course of the LPS response of MDM from 56 individuals (primary data in Table S1) were subjected to network analysis using *BioLayout* as described in “Materials and Methods”. **A** *Sample-to-sample network.* Each sphere represents a sample (individual time point). Edges between them (Pearson correlations of ≥ 0.87) have been omitted for clarity. Note the clear separation of the samples based upon time of incubation (left) but not on disease status (right). **B** *Gene-to-gene network.* Each sphere represents a gene. Edges between them (Pearson correlations of ≥ 0.7) have been omitted for clarity and only the main element of the network is shown. Spheres are coloured by their membership of a cluster (MCL inflation value 2.2), Graphs show the averaged expression of genes in key clusters; colours of the columns match those in the network graph. *Y* axis—gene expression in CPM. *X* axis—the samples are ordered by time point and disease status; each column represents a single sample. Upper bar—disease status (red: affected with Crohn’s disease or ulcerative colitis; black: unaffected sibling of an affected individual; blue: healthy donor with no family history of inflammatory bowel disease). Lower bar—time point (red: 0 h; yellow: 2 h; blue: 7 h; green: 21 h). The average expression profiles for all clusters are provide in Table S2**.** Arrows indicate the position of the cluster in the network. The position of Cluster 70 is not indicated as it forms a small separate element not linked to the largest element shown here

A sample-to-sample network (analogous to a PCA) shown in Fig. 1A (left panel) demonstrates that based upon their transcriptomic profiles, samples from each of the time points group together, with a transition across the network from pre-LPS to 21 h post-LPS reflecting the known progressive temporal profile of the response (Baillie et al. 2017). There was no class separation at any time point between affected and unaffected individuals or healthy donors (Fig. 1A**,** right panel) indicating that any direct effects of disease status and/or ongoing treatments on the transcriptome are not sustained following monocyte cultivation in CSF1. The lack of any global distinction in LPS responsiveness is clearly incompatible with a proposed disease-associated hyper-responsiveness to LPS-stimulated TLR4 activation in CD patient macrophages (Papoutsopoulou et al. 2019).

### Gene-to-gene network defines the conserved temporal cascade of the response to LPS

Figure 1B shows the main elements of the gene-to-gene network graph based on the entire dataset. The MCL method groups sets of genes that share patterns of transcriptional regulation and are therefore correlated with each other. In the 3-dimensional network graph the nodes (transcripts) within a cluster are given the same color. For each cluster, an average expression profile indicates the overall pattern of expression shared by the transcripts within that cluster. The histograms surrounding the network graph show the average expression of genes within selected clusters discussed below; samples are ordered based upon disease status and time. Consistent with Fig. 1A**,** there is no cluster that distinguishes affected from unaffected individuals. Table S2 shows the gene lists for each cluster alongside the average gene expression profiles of additional clusters.

Clusters 1, Cluster 4, and Cluster 5 provide a quality control for the datasets. Cluster 1 contains 1903 transcripts that were expressed constitutively and relatively invariant between the 56 individuals, with a small decline with time in response to LPS. This cluster comprises mainly house-keeping genes, but also includes macrophage-enriched transcription factors (*SPI1, CEBPA/B*) and surface markers (*ITGAM, ITGAX*). Transcripts in Cluster 5 (391 transcripts (not shown, see Table S2)) were also high-expressed and marginally down-regulated (< two-fold) at 2 and 7 h post LPS. Cluster 4 (401 transcripts) was even less variable between individuals and high-enriched for nuclear-encoded mitochondria-related transcripts and ribosomal protein genes. The consistent detection of these transcripts indicates the robustness of the data and provides a reference point for much greater variation amongst other transcripts.

Cluster 70 (17 transcripts) contains all of the mitochondrial-encoded transcripts, clustered because of the extensive and correlated variation between individuals. This observation is consistent with evidence for high levels of variation in expression of these transcripts in human populations (Wang et al. 2014). This cluster provides a positive control for the detection of known variation.

Cluster 12 (146 transcripts) is a macrophage differentiation cluster that validates MDM as a model for gut resident macrophages. The transcripts in this cluster were shown previously to be CSF1-inducible in MDM compared to freshly-isolated monocytes (Baillie et al. 2017). They were high-expressed in MDM and down-regulated by LPS. This cluster includes transcription factors *ETV5, MAFB*, *MEF2C, NFXL1*, *RB1* and multiple macrophage-enriched differentiation markers and cell surface receptors (*AIF1, CD163, CD28, CD302, CD4, CLEC10A, CSF1R, FOLR2, GAS6/7, GPR34, GSDMA, INPP5D, MERTK, MPEG1, MRC1, TLR7, TYROBP, STAB1, TNFRSF11A, TYROBP, VSIG4*). Their correlated expression indicates that there is significant variation in CSF1-induced differentiation of monocytes amongst individuals independent of disease status. ETV5 (Gazova et al. 2020), MAFB (Sarrazin et al. 2009) and MEF2C (Schuler et al. 2008) have each been identified as regulators of monocyte–macrophage differentiation, and variation in their expression (in each case > ten-fold amongst individuals in the 0 time samples) likely contributes to the co-regulation of genes within Cluster 12. Surface CD14, which varied 14-fold at the mRNA level amongst individuals, has been used as a subset marker in studies of intestinal macrophage function (Kamada et al. 2009). *CD14* varied independently of Cluster 12. *CD14* mRNA is high-expressed (along with all the mRNA mentioned above) in published single cell RNA-seq data for human colon (Domanska et al. 2022; Elmentaite et al. 2021). Every one of the differentiation markers in Cluster 12 is also present within a gut resident macrophage signature identified in single cell-RNA-seq analysis of IBD lesions (Hegarty et al. 2023; Martin et al. 2019).

Clusters 2, 3, 9 and 11 group transcripts that share temporal patterns of regulation in the response to LPS. Transcripts in Cluster 11 (161 transcripts) peaked at 2 h and then declined rapidly. They include genes encoding the key feed forward activators, *TNF, IFNB1* and *IFNL1*, transcriptional regulators (e.g. *ATF3, FOSL1, KLF6, KLF7, MYC, NFATC1, NFE2L2, NFIL3, NFKBIZ*, *NR4A3*) and many negative feedback regulators (e.g. *DUSP1, GPR183, NFKBIA, OTUD1, TNFAIP3* and *ZFP36L2*) that serve to constrain the response (Baillie et al. 2017). Transcripts within Cluster 9 (209 transcripts) were also induced by 2 h, but the expression declined less rapidly and was retained at 7 h. This cluster includes additional transcription factors *IRF1, IRF8*, several interferon target genes (*IFIT2*), inducible cytokines (*IL1B, IL12A*) and chemokines (*CXCL1, CCL3, CCL4*).

Cluster 2 (819 transcripts) groups the transcripts that were maximally induced at 7 h post-stimulation and declined by 21 h. It includes inducible transcription factors (*BATF, BATF2, BATF3, IRF9, NFE2L3, STAT2,3,4*) and further interferon target genes (e.g. *IFIT1, ISG15, MNDA, MX1, OAS1*)*.* Similarly, Cluster 3 (445 transcripts) contains transcripts that were inducible by 7 h but continued to increase, being highest at 21 h in the response. This cluster also contains transcripts encoding transcriptional regulators (*CEBPB, IRF6, RELB, STAT1*) and a distinct cohort of chemokines, surface receptors (e.g. *CLEC4E*) and interferon-inducible transcripts including the receptor *IFNAR2* and *IFI6, IFI27, IFITM1/2/3*.

Cluster 13 is related to the cell cycle. Although blood monocytes are predominantly post-mitotic, CSF1 is able to promote proliferation in a subset of cells and this response is inhibited by LPS (Clanchy et al. 2006). Accordingly, Cluster 13 (104 transcripts) contains the key cell cycle transcription factors (*E2F1/E2F2/E2F8, FOXM1, MYBL2*) and numerous structural and regulatory genes that are induced specifically in S phase and mitosis (Giotti et al. 2019). The expression of transcripts in this cluster was relatively low and down-regulated by LPS, but clustering arises because of the substantial variation between individuals at the earlier time points.

To test the relationship between coexpression clusters and IBD disease susceptibility loci we performed gene-set enrichment analysis using MAGMA (Multi-marker Analysis of GenoMic Annotation) (de Leeuw et al. 2015). Amongst the clusters with > 100 transcripts, we detected significant (p_MAGMA_ < 0.05) heritability enrichment in IBD for three clusters, namely Cluster 2 (ß_MAGMA_ = 0.081, p_MAGMA_ = 0.016), Cluster 5 (ß_MAGMA_ = 0.085, p_MAGMA_ = 0.043), and Cluster 9 (ß_MAGMA_ = 0.17, p_MAGMA_ = 0.013).

### Variation in the temporal profile of expression of key cytokine genes

Many pro-inflammatory cytokines produced by macrophages are targets for successful therapeutic intervention in IBD and/or are affected by regulatory variants that are in turn associated with susceptibility to inflammatory disease. As noted above, the majority of transcripts varied by less than two-fold amongst the 50 individuals from IBD families at each of the 4 time points. To identify the most highly variable transcripts, in Tables S1C–F the genes have been ranked in order of maximum/minimum at each time point. This is a relatively simple screen for homozygous loss of expression alleles. It is internally validated by detection of the Y chromosome-specific transcripts (*DDX3Y, EIF1AY, KDM5D, RPS4Y1, USP9Y, UTY, ZFY*) which distinguished male from female individuals and formed a small co-regulated cluster in which females have zero expression, equivalent to a null allele (Cluster 176, Table S2). Note that there is no other sex-specific expression cluster. In unstimulated MDM we detected extreme variation in the level of *CHIT1*, which is high-expressed in MDM. Around 35% of individuals are heterozygous for a 24-base pair duplication in exon 10 of *CHIT1* that results in activation of a cryptic 3’ splice site, generating a mRNA with an in-frame deletion of 87 nucleotides (Boot et al. 1998). The effect of this variant on mRNA expression or stability is not known. Similarly, 12/56 individuals appear to have a loss of expression of *HLA-E.* Tables S1C–F also show the ratio of average expression in affected (AF) versus unaffected (UN) at each time point and average expression in individuals with a positive or a negative polygenic risk score (PRS) (see Table S1A) regardless of disease status. Consistent with the network analysis, there were no significant differences in individual gene expression that distinguish affected from unaffected individuals as a class and there was no significant difference in expression of any transcripts associated with positive PRS.

At each of the three time points post LPS treatment (2 h, 7 h, 21 h) there were around 100 transcripts that varied more than 100-fold in expression between individuals (Table S1D–F). The top 100 hypervariable transcripts at 2, 7 and 21 h included numerous chemokines (*CCL2, CCL4, CCL5, CCL15, CCL19, CCL20, CCL22 CCL3L3, CCL4L2, CCL8, CXCL1, CXCL2, CXCL3,CXCL5, CXCL8, CXCL9, CXCL10, CXCL11*) and cytokines (*CSF2, CSF3, EBI3, IFNB1, TNF, TNFSF10, IL1A, IL1B, IL6, IL12B, IL23A, IL27, IL32*) many of which are considered targets for therapeutic intervention (Camba-Gomez et al. 2022) and all of which were previously associated with condition-dependent eQTL in monocytes (Fairfax et al. 2014). One feature of the LPS response in human MDM that has not previously been reported is the induction of the chemokine receptor, *CCR7* and its ligand, *CCL19.* Both were variable between individuals and not correlated with each other (Table S1D-F). This cocktail of inducible macrophage-derived effectors likely contributes to the formation of tertiary lymphoid tissue that is a feature of IBD (McNamee and Rivera-Nieves 2016). In some cases, the variation reflects temporal differences.

The relative abundance and function of inducible negative feedback regulators of macrophage activation highlighted previously (Baillie et al. 2017) also varied between individuals. For example, the set of high-expressed and hypervariable transcripts in Table S1D–F includes *ACOD1, DUSP1, GPR183, OTUD1, PMAIP1, PTGS2* (aka *COX2*)*, SERPINE1, SOCS3, TNFAIP3, TNFAIP6* and *ZFP36*. These have been described as inflammation suppressor genes in that even heterozygous inactivating mutations in any one of them can lead to spontaneous inflammation (Kondo et al. 2012; Wells et al. 2005).

**Fig. 2:**
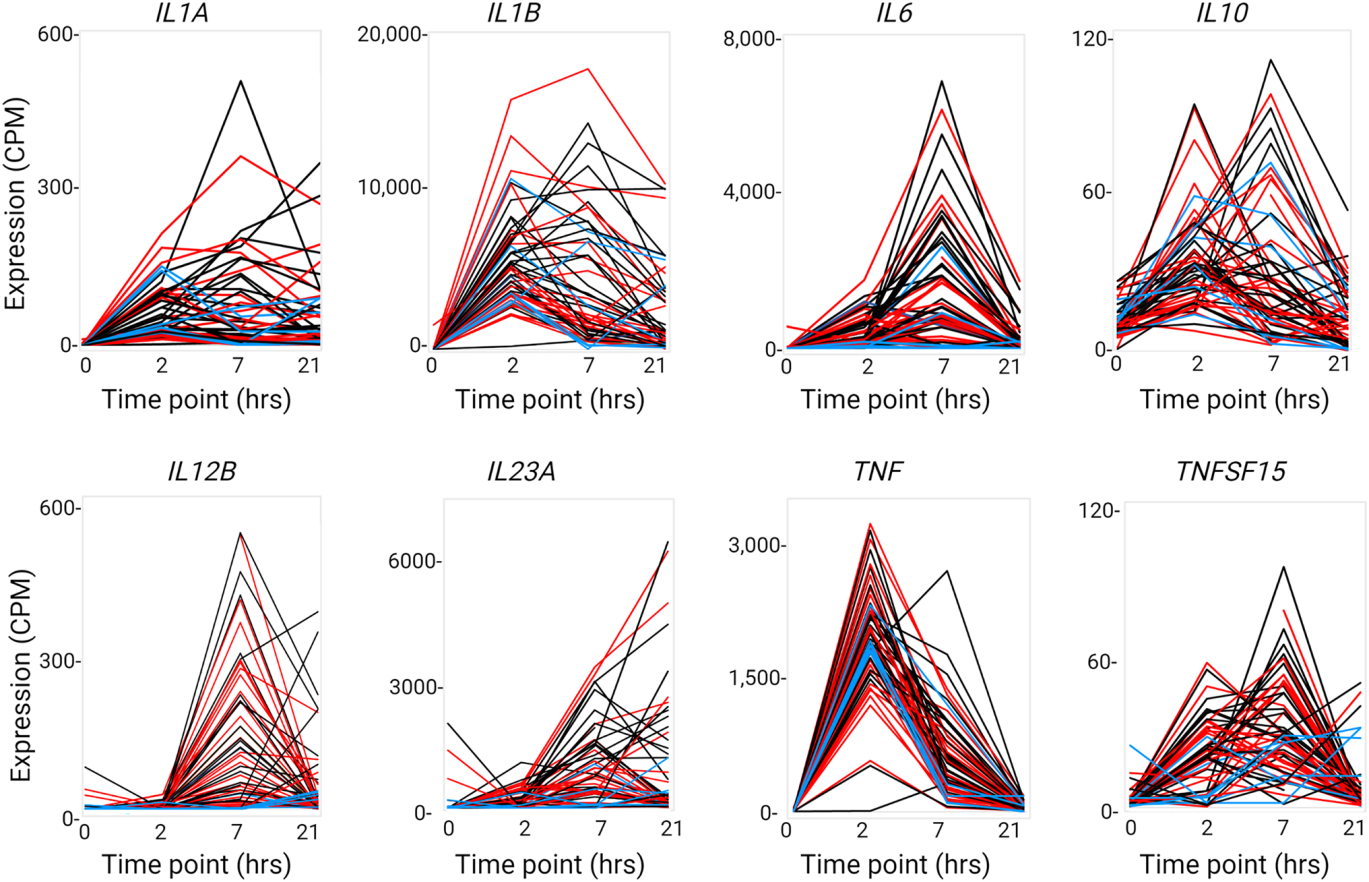
Individual variation in patterns of transcriptional regulation of key cytokine genes in LPS-stimulated MDM. Gene expression profiles for the indicated cytokine genes were extracted from the RNA-seq analysis of the time course of the LPS response of MDM from 56 individuals (primary data in Table S1B–F). Each line joins the 4 time points from a single individual (red: affected with Crohn’s disease or ulcerative colitis; black: unaffected sibling of an affected individual; blue: healthy donor with no family history of inflammatory bowel disease)

To illustrate the nature and extent of the diversity, Fig. 2 shows the time courses of regulation of selected cytokines (*IL1A, IL1B, IL10, IL12B, IL23A, IL6, TNF* and *TNFSF15*) for each of the 56 individuals across the whole dataset, color-coded by disease status. The transient induction of *TNF* with a peak at 2 h and of *IL12B* at 7 h was relatively consistent between individuals apart from a small number of outliers with low or sustained expression (Fig. 2, Table S1C, D). For other transcripts, the temporal pattern as well as the magnitude of the response was more variable. For example, in 9 individuals, *IL6* mRNA peaked at a relatively low level at 2 h then declined, where in the majority, peak expression at a much higher level occurred at 7 h. In the majority (35/56), *IL1B* peaked at 2 h then declined rapidly, but in 21/56 *IL1B* mRNA was sustained or increased further at 7 and 21 h.

**Fig. 3:**
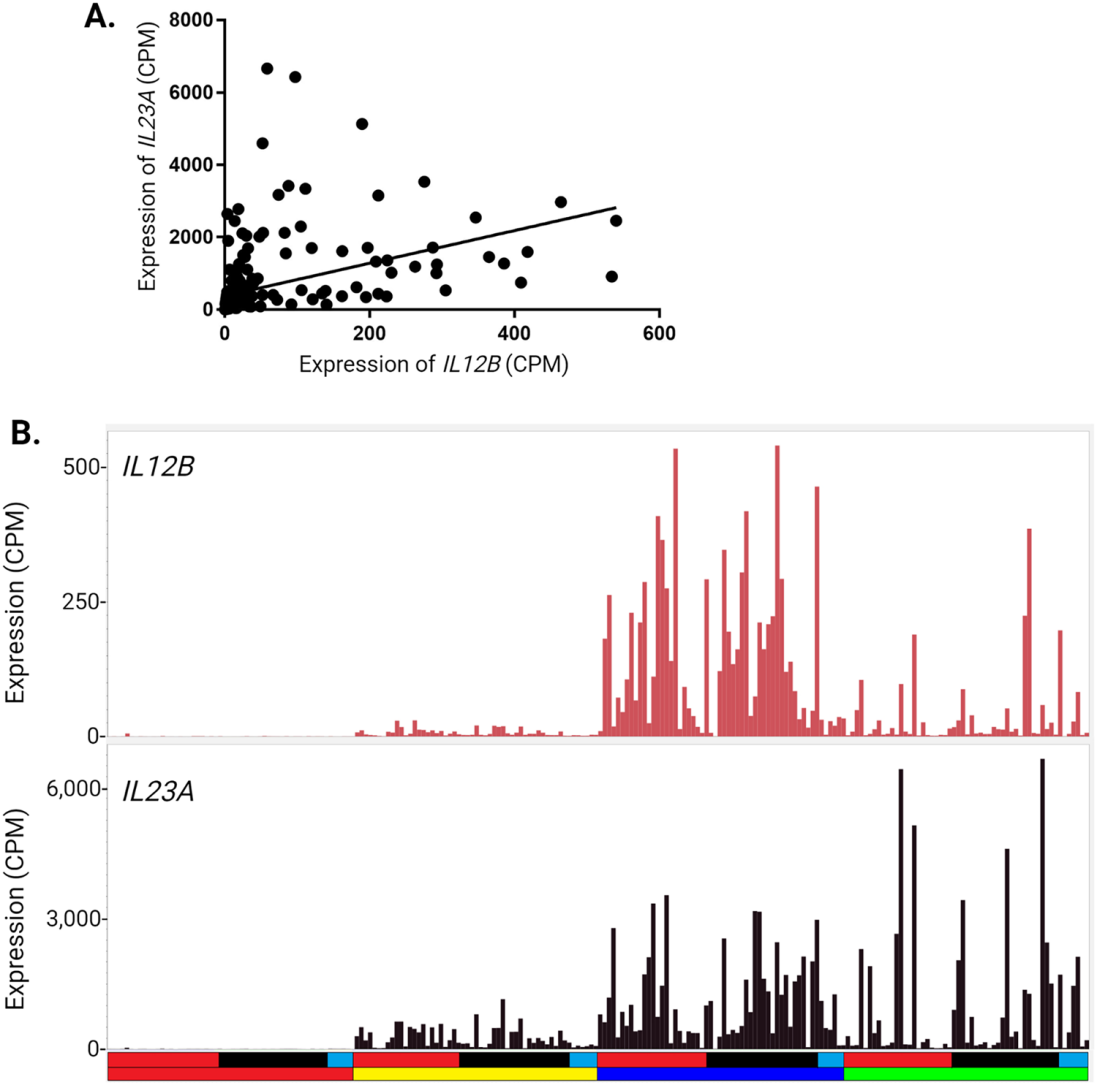
Expression of *IL23A* and *IL12B. IL23A* and *IL12B* encode the two subunits of the heterodimeric cytokine, IL23. Expression of the two genes was extracted from RNA-seq analysis of the time course of the LPS response of MDM from 56 individuals (primary data in Table S1). *A. Correlation between IL23A and IL12B expression.* Graph shows the relationship between the two transcripts across the full dataset (all 4 time points) including the line of best fit. Spearman *r* = 0.8766, *p* < 0.0001. Zero time values are clustered at left. Note the numbers of outliers above (high *IL23A*) and below (high *IL12B*) the line. *B. Patterns of IL23A and IL12B expression across the time course*. *Y* axis—gene expression in CPM. X axis—samples ordered by time point and disease status; each column represents a single sample. Upper bar—disease status (red: affected with Crohn’s disease or ulcerative colitis; black: unaffected sibling of an affected individual; blue: healthy donor with no family history of inflammatory bowel disease). Lower bar—time point (red: 0 h; yellow: 2 h; blue: 7 h; green: 21 h)

IL23, a dimeric protein comprised of IL12B and IL23A, is a therapeutic target in IBD (Parigi et al. 2022; Sewell and Kaser 2022). Figure 2 shows that *IL23A* and *IL12B* were each strongly induced by LPS but with distinct temporal profiles. In the cluster analysis, *IL23A* forms part of the small hypervariable Cluster 118 (10 transcripts) whereas *IL12B* was not correlated with any other transcript at the threshold of *r* = 0.7. This observation raises the possibility that some individuals are IL23-deficient through a failure to coordinate the expression of the two subunits. Figure 3A shows that the expression of the genes encoding the two subunits is significantly correlated across the whole dataset. However, there are some individuals where one gene is very highly expressed whilst the other is low (Fig. 3B). In addition, the peak level of expression of *IL23A* is an order of magnitude higher than expression of *IL12B.* IL23A may also form dimers with EBI3 to produce an alternative effector of the receptor IL23R, although there is some doubt as to whether this occurs in humans (Ecoeur et al. 2020). Unlike *IL12B* and *IL23A*, *EBI3* forms part of the late response Cluster 3 and was amongst the most highly-induced transcripts with relatively little variation amongst individuals. EBI3 is also a dimer partner with IL27A to form IL27. *IL27A* mRNA was also LPS-inducible and varied greatly between individuals, but unlike the dimer partner, it is part of Cluster 2 (peaking at 7 h) and is a known interferon target gene (de Marcken et al. 2019).

### Variation in gene expression in unstimulated macrophages

Table S1C highlights the transcripts that are hypervariable in unstimulated MDM. The *CSF1* gene is of particular interest. Consistent with the proposed role of CSF1R signalling in intestinal macrophage development, *CSF1* and the alternative agonist *IL34* are both high-expressed in human ileum and colon and further increased in IBD lesions (Zwicker et al. 2015). In humans, *CSF1* mRNA is not detectable in freshly-isolated monocytes but is induced during monocyte–macrophage differentiation (Baillie et al. 2017). *CSF1* but not *IL34*, was high-expressed in unstimulated cells in most individuals and further-induced around threefold by LPS at 2 h. Expression varied independently of other cytokines and *CSF1* formed part of a small cluster with *LIF* and *OCSTAMP* (Cluster 302, Table S2). A relatively common SNV (rs484959) at the *CSF1* locus is linked to Paget’s disease (Albagha et al. 2010) but this SNV was not associated with the variation in *CSF1* expression in MDM in the eQTL analysis (see below). Transcripts encoding the three subunits of complement component C1q (*C1QA, CIQB, C1QC*) are also undetectable in monocytes and high-expressed in MDM. C1q has multiple functions in autoimmunity and in resistance to infection (Lu et al. 2008). Each *C1Q* transcript varied more than tenfold between individuals. *C1QA* and *C1QC* were correlated with each other (Cluster 443, Table S2) at *r* > 0.75. *C1QB* was correlated at *r* = 0.41 (with *C1QC*) and *r* = 0.54 (with *C1QA*). Potter et al. (1979) provided evidence of increased synthesis and catabolism of C1q in IBD patients. An IBD susceptibility locus, defined by rs12568930, is located at Chromosome 1:22,375,738. The nearest gene is *ZBTB40,* but the three C1Q genes lie within the genomic interval, approximately 0.3 Mb from the SNV (Baillie et al. 2017), so this variation could be disease-relevant.

### Macrophages express protein-coding genes associated with IBD susceptibility

Based upon mass scale genome and exome sequencing in thousands of individuals, Sazonovs et al. (2022) confirmed known and identified a number of new coding variants apparently enriched in IBD patients. The pattern of expression and function of transcripts containing these newly-identified coding variants in monocytes and macrophages is shown in Table 1. With only 4 exceptions (*FUT2, IL32R, HGFAC*, *IRGM*) we confirmed that all IBD-associated genes with coding variation identified through genome sequencing were expressed and regulated during monocyte differentiation or activation in our data.

**Table 1:** Macrophage-related function of candidate IBD susceptibility genes with coding variation

Gene name	Function/pathway	Comments	References
*NOD2*	CARD15. Pathogen pattern recognition	Several coding variants *NOD2* mRNA high in monocytes, down-regulated by CSF1 Co-regulated with neighbouring genes *SNX20* and *CYLD*	Keestra-Gounder and Tsolis (2017), Tang et al. (2019)
*ATG16L1*	Autophagy	T300A coding variant. Mechanism/function still unclear. Widely-expressed. Neighbouring *INPP5D* (SHIP1) gene high in monocytes, down-regulated by CSF1	Chesney et al. (2021), Fernandes et al. (2018)
*SLC39A8*	Zinc transporter (ZIP8)	Monocyte/macrophage specific. Highly-inducible by LPS Note adjacent *NFKB1*, also inducible by LPS. Highly variable expression	Hall et al. (2021), Kalita et al. (2018)
*TYK2*	Tyrosine kinase	Broad expression. Neighbouring gene *ICAM3* high in monocytes, down-regulated by CSF1, implicated in IBD	Bernstein et al. (1997)
*IFIH1*	MDA5. Anti-viral defense	Strongly induced by LPS in monocytes and MDM. Variable expression. Link to viral triggers?	Adiliaghdam et al. (2022), Zervou et al. (2022)
*LRRK2*	Multifunctional kinase. PARK8/Dardarin/RIPK7	High in monocytes and granulocytes Down-regulated by CSF1. Regulator of phagosome-lysosome homeostasis	de Guilhem de Lataillade et al. (2022), Eguchi et al. (2018), Lee et al. (2020)
*SLAMF8*	Cell surface receptor (CD353)	Undetectable in monocytes, induced by CSF1. Inhibitor of reactive oxygen production & inflammation	Romero-Pinedo et al. (2022), Zeng et al. (2020)
*PLCG2*	Phospholipase/eicosanoid regulation	Coding mutants in autoinflammatory diseases. Constitutive in monocytes and MDM. Downstream regulator of CSF1 signals	(Jing et al. (2021)
*CARD9*	Signalling adaptor protein	High in monocytes. Repressed by CSF1 and by LPS. Regulation of *IL1B* and inflammasome activation	Drummond et al. (2022)
*ADCY7*	Adenylate cyclase	Monocyte–macrophage specific promoter. Regulates LPS responsiveness. Coding variants associated with ulcerative colitis	Duan et al. (2010), Luo et al. (2017)
*GPR65*	pH-sensing receptor	High in monocytes and granulocytes, Down-regulated by CSF1. Regulator of inflammation	Tcymbarevich et al. (2019)
*SMAD3*	Transcription factor	High in monocytes, down-regulated by CSF1. Regulator of inflammation	Fowler et al. (2014)
*IL10RA*	IL10 receptor	High in monocytes, down-regulated by CSF1 but high-induced by LPS and variable. Mutation in early-onset IBD	Kelsen et al. (2015)
*PTAFR*	Platelet activating factor receptor	High in monocytes, down-regulated by CSF1	Liu et al. (2020)
*TAGAP*	RhoGTPase activating protein	High in monocytes, down-regulated by CSF1, induced by LPS, highly variable. Shared risk with celiac disease	Festen et al. (2011)
*IRGM*	Putative regulator of autophagy	*IRGM* mRNA is not detectable in any tissue. Neighbouring *TNIP1* (ABIN3) high-expressed in MDM, induced further by LPS. TNIP1 is a binding partner of TNFAIP3	Zhou et al. (2021)
*RELA*	NFkB transcription factor	Major transducer of LPS signaling. High in monocytes and MDM, further induced by LPS	Papoutsopoulou et al. (2019)
*DOK2*	Adaptor protein, regulator of tyrosine kinase signals	High in monocytes, down-regulated by CSF1. Further repressed by LPS	Shinohara et al. (2005)
*CCR7*	Chemokine receptor	Induced around 500-fold by LPS by 21 h. Very variable between individuals. Major ligand, CCL19, also induced > 1000-fold. Generation of tertiary lymphoid structures	Koscso et al. (2020)

Genes above were shown to have coding sequence variants significantly linked to IBD susceptibility in large-scale whole genome sequencing analysis of affected individuals and controls (Sazonovs et al. 2022). Comments relate to expression in monocytes and macrophages in the current dataset and/or FANTOM5 CAGE data analysed previously (Baillie et al. 2017). Selected references highlight known functions of gene of interest and/or neighbouring genes in macrophages or inflammation

IRGM has been proposed to regulate autophagy and to interact with NOD2 and ATG16L1 to control anti-microbial defense (Chauhan et al. 2015). The issue with coding variation in IRGM is that there is no evidence of expression of *IRGM* mRNA in the RNA-seq data generated herein, or in any tissue in large public datasets (Barbeira et al. 2021; Forrest et al. 2014). However, enhancers active in monocytes/macrophages were detected throughout the *IRGM* locus and flanking DNA (Arner et al. 2015). As previously discussed (Baillie et al. 2017) the neighbouring *TNIP1* gene (also known as *ABIN3*) encodes a more obvious regulator of inflammation located in the *IRGM* genomic region and regulatory variation in this gene has been detected in patients with systemic lupus erythematosus (Raj et al. 2016). In our data, unlike *IRGM, TNIP1* mRNA was high-expressed in MDM, further induced > ten-fold by LPS and highly variable amongst individuals (Table S1C–F).

### eQTL analysis

**Fig. 4:**
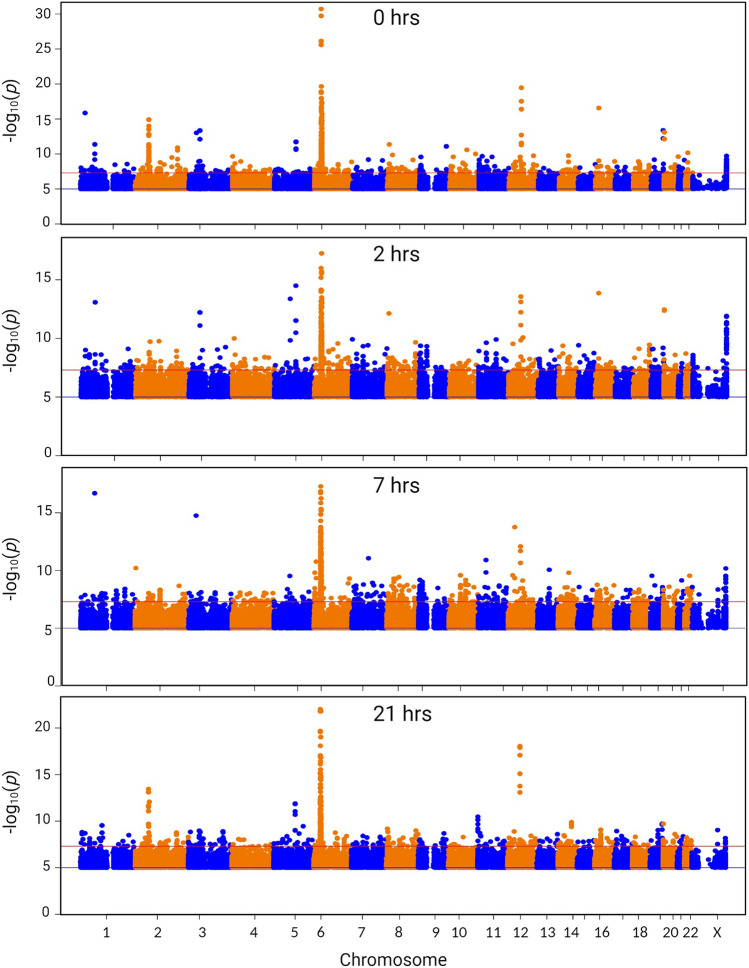
Expression QTL analysis of the response of MDM to LPS. Gene expression profiles from RNA-seq analysis of the time course of the LPS response of MDM from 56 individuals (primary data in Table S1) were correlated with SNV genotype as described in “Materials and methods”. *Y* axis—*p* value (− log10) of the correlation coefficient for the SNV genotype. Primary data for *cis*-acting variants are provided in Table S3. *X* axis—chromosome number and position for each analyzed SNV. Colours alternate with odd numbered chromosomes in blue and even numbered chromosomes in orange. Note that peaks include multiple *p*-values for single variants which are associated with multiple genes within the genomic interval

Previous studies in larger cohorts have identified allele-associated variation in regulated gene expression in monocytes and MDM (Brandt et al. 2021; Fairfax et al. 2014; Fan et al. 2020; Kim-Hellmuth et al. 2017, 2020; Rotival et al. 2011). Network analysis of the monocyte data for > 300 subjects (Fairfax et al. 2014) using *BioLayout* identified *trans*-acting impacts of variation in expression of *IFNB1* and downstream transcription factors *IRF7* and *IRF9* (Hume and Freeman 2014). The relatively small population analyzed herein, and the obvious and deliberate relatedness, is not optimal for any quantitative genetic analysis, including eQTL (Sul et al. 2018). However, given the magnitude and prevalence of the variation within the IBD families in Tables S1C–F we speculated that *cis*-acting variation of large effect associated with specific genes might still be detectable. To test this possibility, we performed eQTL analysis based upon the ImmunoArray BeadChip v2.0 genotypes. Figure 4 shows the Manhattan plots for each of the 4 time points and Table S3 contains all *cis* associations with a *p* value of < 10^–5^, a false discovery rate < 0.01. A major peak of *cis*-acting variation was detected over the HLA region of chromosome 6 at every time point. The *cis-*acting variation is linked to variable expression of Class I and Class II HLA genes, as well as genes within the HLA region, including *CLIC1, GPSM3, MICA, RNF5*, *TRIM26* and *ZFP57.* Notably although *TNF* is within the HLA complex, there was no eQTL associated with *TNF.* A significant eQTL was associated with an extended haplotype at the *C2Orf74/USP34* locus on chromosome 2. This is a known IBD susceptibility locus (Ellinghaus et al. 2016). eQTL analysis of peripheral blood detected SNV association with expression of multiple genes in the region including *PUS10, REL, AHSA2* and *KIAA1841* in addition to *C2Orf74* and *USP34* (Ellinghaus et al. 2016). An additional eQTL peak reproducible at each time point was on chromosome 12, associating multiple variants across a 100 mb interval with expression of *RPS26* (see Table S3). *Cis*-acting regulation of *RPS26* expression was reported previously in the context of type 1 diabetes (Plagnol et al. 2009) and verified in a large monocyte dataset (Rotival et al. 2011) but the biological significance is unclear.

An eQTL peak on chromosome 19 was detected at all timepoints associated with expression of the members of the inhibitory leukocyte Ig-like receptor family (*LILRB1-4*), *NRLP2* and *LENG8.* This was identified as an IBD susceptibility locus by GWAS (Jostins et al. 2012) and expression of these transcripts is strongly-enriched in monocytes and macrophages (Baillie et al. 2017). An eQTL peak on chromosome 20 was associated with *CD40*, and included a known SNV, rs4810485, associated with IBD in a large Spanish cohort (Blanco-Kelly et al. 2010). Finally, also on chromosome 20 we detected an eQTL associated with *SIRPB1.* A recent study reported association between a relatively uncommon C terminal frame-shift variant, the level of expression of *SIRPB1* and Crohn’s disease in Han Chinese (Tang et al. 2023).

We detected few conditional eQTL, with specific effects at 2 h, 7 h or 21 h at the stringent FDR threshold chosen and none in which the affected transcript was high-expressed in MDM. One example, an eQTL for *IL18R1* was detected at 2 h and 7 h, associated with SNVs across *IL18RAP/IL1RL1/IL18R1* locus including IBD risk variant rs917997. Hedl et al. (2014) suggested that autocrine IL18 stimulation in MDM and regulated cytokine secretion was associated with rs917997 genotype. However, *IL18R1* and *IL18RAP* were expressed at < 2 CPM in MDM in our data and in FANTOM5 data, although the entire region contains numerous monocyte-specific enhancers (Forrest et al. 2014). *IL18* was expressed constitutively in MDM and was not associated with an eQTL.

### SNV association with expression of specific genes

To further test the relationship between local genotype and the highly variable expression of specific transcripts we examined SNV associations at selected candidate gene loci implicated in IBD pathology and where we identified sufficient homozygotes of the alternative genotypes.

**Fig. 5:**
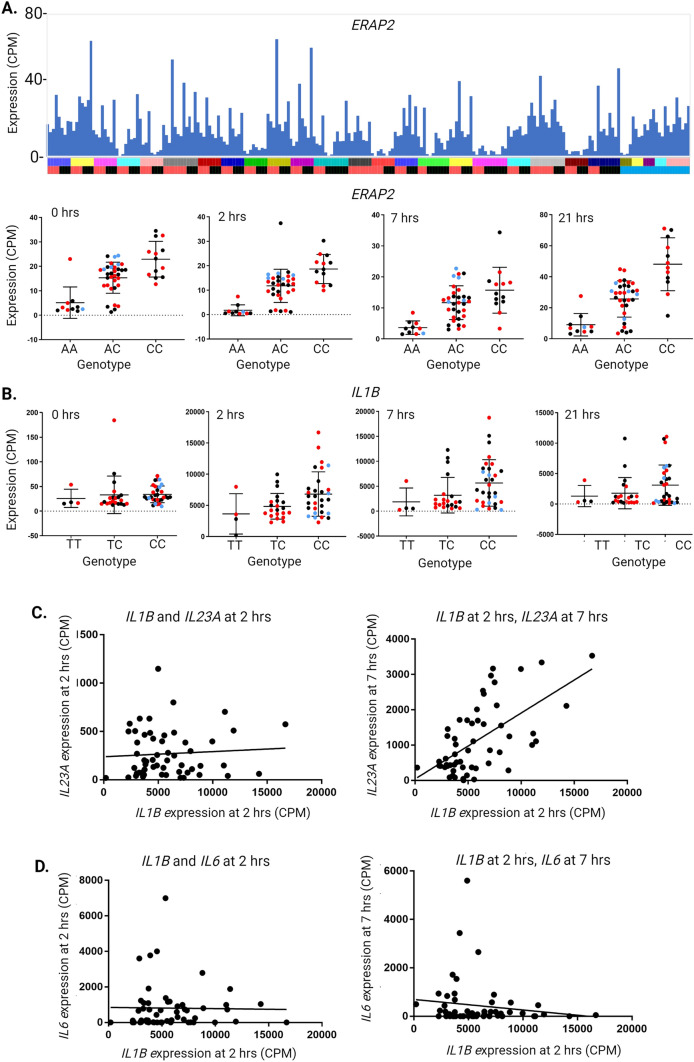
Relationship between SNV genotype and expression of key genes. Expression of the genes indicated was extracted from RNA-seq analysis of the time course of the LPS response of MDM from 56 individuals (primary data in Table S1) and SNV genotype was determined as described in “Materials and methods”. **A** *Expression of ERAP2.* Histogram in the upper panel shows the levels of expression of *ERAP2* for each individual across the four time points. *Y* axis—expression in CPM. *X* axis—individuals and time points. Each column represents one sample and the four time points for each individual are adjacent to each other and to other family members. Upper bar—family (arranged as in Table S1). Lower bar—disease status (red: affected with Crohn’s disease or ulcerative colitis; black: unaffected sibling of an affected individual; blue: healthy donor with no family history of IBD). Graphs in the lower panel show the expression values for each genotype at *ERAP2* SNV rs1056893 for the four time points. Colour of dots indicates disease status (red: affected with Crohn’s disease or ulcerative colitis; black: unaffected sibling; blue: healthy donor with no family history of IBD). Kruskal–Wallis test showed a highly significant difference amongst genotypes for all time points (*p* < 0.0001), with the AA genotype showing lower expression than the CC genotype and the heterozygous genotype being intermediate. **B** *Expression of IL1B.* Graphs show the expression values for each genotype at *IL1B* SNV rs1143634 for the four time points. *Y* axis—expression (CPM). *X*-axis—genotype. Colour of dots indicates disease status (red: affected with Crohn’s disease or ulcerative colitis; black: unaffected sibling of an affected individual; blue: healthy donor with no family history of IBD). Kruskal–Wallis test showed no difference amongst genotypes for 0 h (*p* = 0.17) and 21 h (*p* = 0.39). There was a small difference amongst genotypes at 2 h (*p* = 0.03) and 7 h (*p* = 0.02), with the TT genotype showing lower expression than the CC genotype. **C** *Correlation between IL1B and IL23A expression.* Left panel shows the relationship between *IL1B* and *IL23A* expression at 2 h. Spearman *r* = 0.05577, *p* = 0.68. Right panel shows the relationship between expression of *IL1B* at 2 h and *IL23A* at 7 h. Spearman *r* = 0.5573, *p* < 0.0001. **D** *Correlation between IL1B and IL6 expression.* Left panel shows the relationship between *IL1B* and *IL6* expression at 2 h. Spearman *r* = 0.08593, *p* = 0.53. Right panel shows the relationship between expression of *IL1B* at 2 h and *IL6* at 7 h. Spearman *r* = − 0.119, *p* = 0.39

***ERAP2.**** ERAP2* lies within a genomic interval associated with IBD susceptibility (Baillie et al. 2017). The locus contains three related genes, *ERAP1, ERAP2* and *LNPEP,* that encode aminopeptidases involved in antigen processing (Paladini et al. 2020). Promoter analysis by the FANTOM5 Consortium revealed that *ERAP2* is contained within an intron of *ERAP1* which shares a bidirectional promoter with *LNPEP* (Forrest et al. 2014). The eQTL analysis identified a strong *cis*-eQTL affecting expression of *ERAP2* (Table S3). Expression was associated with SNVs within the gene as well as the neighbouring *LNPEP* gene. There are two major *ERAP2* variants with almost equal frequency in human populations (Klunk et al. 2022) one of which encodes a splice isoform that introduces premature stop codons leading to nonsense-mediated decay (NMD). Klunk et al. (2022) provided evidence that the functional allele was favoured by pathogen selection in survivors of the black death pandemic, caused by the bacterium *Yersinia pestis*, and suggested a consequential increase in susceptibility to inflammatory disease including IBD. Figure 5A (upper panel) shows that a subset (13/56) of our cohort has very low expression *ERAP2* in MDM. Analysis of expressed SNVs revealed that the individuals that lacked *ERAP2* expression were indeed generally homozygous for the rs1056893 variant but there was no association with disease status (Fig. 5A**,** lower panel). *ERAP2* was also identified in eQTL analysis of peripheral blood (Ellinghaus et al. 2016). Based upon regulated expression in monocytes and macrophages we previously argued that neighbouring genes are more likely candidate genes underlying IBD susceptibility associated with the *ERAP2* locus (Baillie et al. 2017). *ERAP1* and *LNPEP* are more highly and consistently expressed in MDM than *ERAP2.* Extensive protein-coding polymorphism in each of the genes is associated with susceptibility to multiple inflammatory diseases including IBD (Ellinghaus et al. 2016; Paladini et al. 2020).

***IL1B.**** IL1B* is of particular interest because of its implication in IBD pathology (Aschenbrenner et al. 2021) and because LPS-inducible expression is at least ten-fold lower in MDM compared to freshly-isolated monocytes (Baillie et al. 2017). Hence, extreme high expression of *IL1B* in response to LPS may be considered an indication of failure of CSF1-induced differentiation. SNV rs1143634 encodes a synonymous amino acid substitution in IL1B. Figure 5B shows level of expression of *IL1B* mRNA at each time point for each genotype at this SNV. The distribution of peak expression values is highly skewed but there was a significant correlation between the presence of the minor (T) allele and lower expression at 2 and 7 h post LPS treatment. Aschenbrenner et al. (2021) proposed that IL1B provides an autocrine signal regulating expression of another therapeutic target IL23A (Schmitt et al. 2021; Sewell and Kaser 2022) by stimulated monocytes. Consistent with that view, *IL23A* was induced later in the LPS response (peak at 7–21 h; Fig. 2) and expression of *IL23A* at 7 h was significantly correlated with *IL1B* at 2 h (Fig. 5C). To test the specificity of that interaction and to identify additional IL1B targets, we used *BioLayout* to generate a Spearman correlation matrix between *IL1B* at 2 h and all other genes at 7 h with a minimum correlation coefficient of 0.6 (compared to 0.55 for *IL23A*). The results are shown in Table S4. More than 300 correlated transcripts were identified, the most significant being *IL1A, CXCL1, CXCL2, CXCL3, CXCL8* and *NLRP3*.

***IL6.*** IL6 is another therapeutic target in many inflammatory diseases (Tanaka et al. 2014). *IL6* mRNA at 7 h varied > 100-fold between individuals (Fig. 2). The *IL6* locus contains a common promoter variant (–174G/C, rs1800795) which has been associated with circulating IL6 and with variation in responsiveness to LPS and other stimuli (Tanaka et al. 2014). Unfortunately, this SNV is not on the ImmunoArray, but there were four informative *IL6*-associated SNVs in LD with each other. Figure S2A shows the lack of any relationship between SNV genotype and *IL6* mRNA expression level at any of the timepoints in our cohort. Importantly, by contrast to the relationship between *IL1B* and *IL23A,* there was no correlation between *IL6* mRNA at any time point and the level of *IL1B* at 2 h (Fig. 5D). Expression of the inducible anti-inflammatory cytokine, *IL10,* was similarly unrelated to SNV genotype at the locus (Fig. S2B).

**Interferon and IRFs*.*** Fairfax et al. (2012) highlighted a *trans*-acting transcriptional network involving members of IRF family, notably *IRF2*, underlying eQTL in monocytes, and multiple chemokines of the CXCL family as targets. It is notable that *IRF7,* which also formed part of an IFN-inducible regulon in monocytes responding to LPS (Hume and Freeman 2014; Knight 2013), was not found in any cluster in the Network analysis (Fig. 1B, Table S2). Indeed, by contrast to the induction reported in monocytes, *IRF7* was induced by LPS in MDM derived from only 9/56 individuals. On the other hand, *IRF1* (which may be affected by an autoimmune disease risk variant in monocytes (Brandt et al. 2021)) was strongly induced by LPS to a similar extent in MDM in all individuals. *IRF1, 2, 3, 4, 5, 7, 8, 9* were all expressed constitutively in MDM and further induced by LPS, likely in part due to endogenous IFNB1. The *IFNB1* gene was induced transiently at 2 h and high-variable between individuals (Table S1D). To confirm this relationship, we used *BioLayout* to generate the relationship matrix between *IFNB1* at 2 h and all other genes at 7 h. The results are shown in Table S4 at three Spearman *r* values *r* > 0.6, *r* > 0.7 and *r* > 0.73. They are consistent with the view that extensive variation in expression of IFN target genes is controlled significantly by the level of expression of endogenous IFNB1. A functional variant in a distal enhancer within the *PTPLAD2* locus on chromosome 9 has been implicated in transcriptional regulation of *IFNB1* in human monocytes (Assouvie et al. 2020) but there are no informative SNPs in the region on the ImmunoArray. *IRF2* expression was not highly-variable in stimulated MDM and was not correlated with SNV genotype (Fig. S2C). *IRF1* was most highly-expressed and LPS-inducible and varied fourfold at 2 h and 16-fold at 7 h between individuals. *IRF1* lies within the IBD5 locus at 5q31, where there is extensive LD (Onnie et al. 2006). Other candidate genes in this region that were regulated by CSF1 and/or LPS in our dataset include *SLC22A4, SLC22A5* and *CSF2;* the latter is hypervariable in expression in response to LPS (Table S1E)*.* However, by contrast to the reported association between an IBD-associated variant and *IRF1* expression in LPS-stimulated primary monocytes (Brandt et al. 2021), we found no correlation between SNV genotype and *IRF1* mRNA at either time point. As noted above, induction of *IRF7* in MDM by LPS appeared to be an all-or-nothing response and there was a significant correlation between genotype and expression at 2 and 7 h (Fig. S2D). The only member of the IRF family detected at multiple time points in the eQTL analysis was *IRF5* on chromosome 7 (Table S3).

**CXCL chemokines.** Neutrophil chemoattractant chemokines of the CXCL family were all inducible by LPS and hypervariable. One theory of IBD pathogenesis suggests that disease arises from a failure of neutrophil recruitment (Segal 2018). *CXCL1, CXCL2, CXCL3* and *CXCL8* are within a common locus on chromosome 4, containing multiple regulated intergenic enhancers (Baillie et al. 2017). As noted above, their expression at 7 h was strongly correlated with the level of *IL1B* at 2 h. We detected no relationship between local SNV genotype and expression of *CXCL1* (Fig. S3A) but consistent with their colocation on the genome and likely shared transcriptional regulation, the *CXCL1, CXCL2, CXCL3, CXCL8* chemokine transcripts were high-correlated with each other (as well as *IL1B*) and substantially less with other CXCL chemokines. Similarly, the co-located *CXCL9, CXCL10* and *CXCL11* transcripts, targets of IFN signalling (Table S4) were most correlated with each other (Fig. S3B) further exemplifying the robustness of the data.

In overview, *ERAP2* provides a definitive positive control for the detection of *cis-*acting SNV association with expression of specific transcripts in our dataset. By contrast, aside from the HLA region and examples cited above we find little evidence to indicate an association between common SNVs and the extreme variation of expression of specific transcripts detected in MDM.

### Association of the regulation of MHC gene expression with HLA haplotypes

**Fig. 6:**
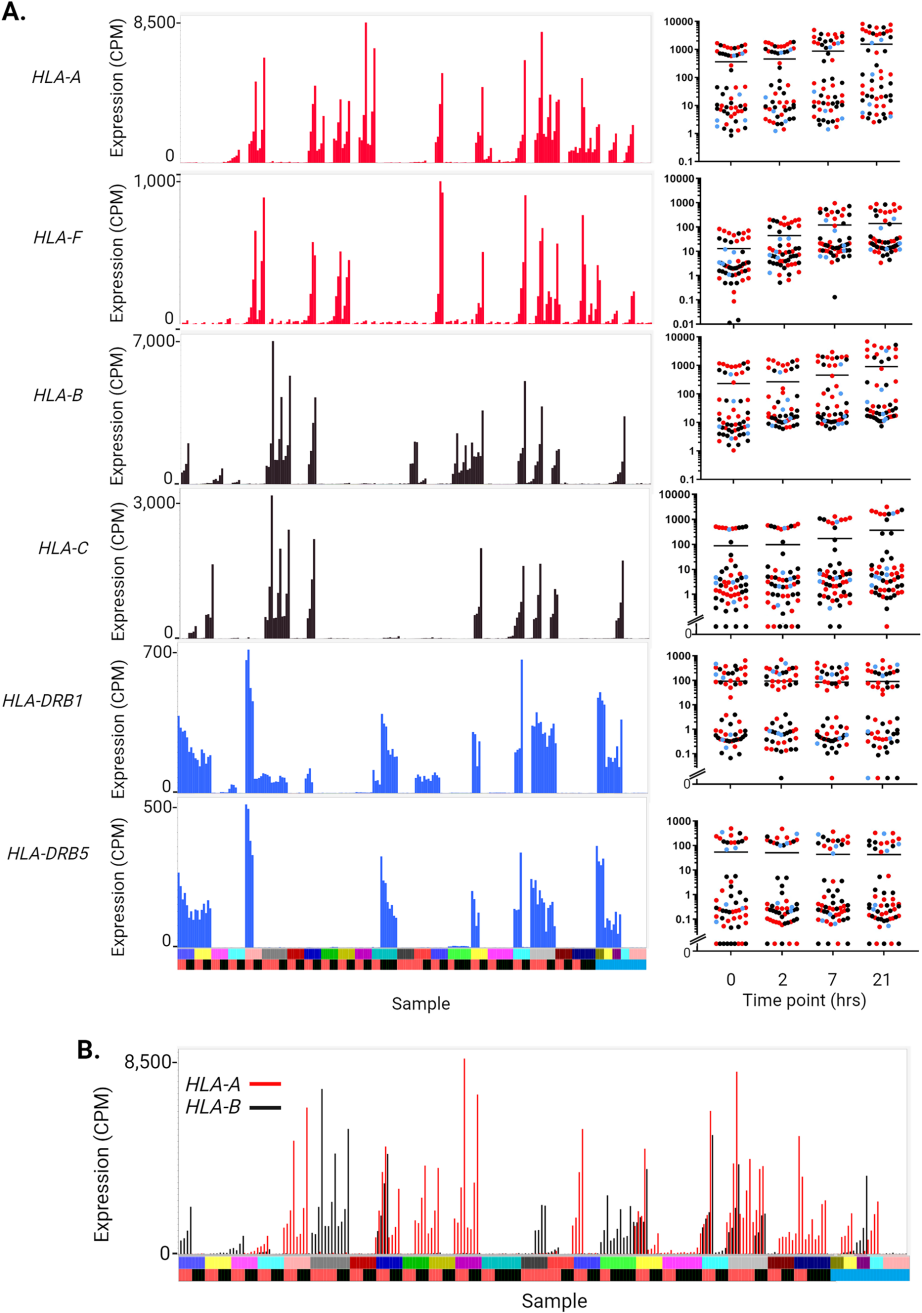
Expression of MHC genes in IBD patients and families. Expression of the Class I and Class II MHC genes was extracted from RNA-seq analysis of the time course of the LPS response of MDM from 56 individuals (primary data in Table S1). (**A**)* Expression of MHC genes.* Histograms on the left show the levels of expression for each individual across the four time points. Expression of *HLA-A* and *HLA-F* (red), *HLA-B* and *HLA-C* (black) and *HLA-DRB1* and *HLA-DRB5* (blue). *Y* axis—expression in CPM. *X* axis—individuals and time points. Each column represents one sample and the four time points for each individual are adjacent to each other and next to other family members. Upper bar: family (arranged as in Table S1). Lower bar: disease status (red: affected with Crohn’s disease or ulcerative colitis; black: unaffected sibling of an affected individual; blue: healthy donor with no family history of IBD). Panels on the right show the distribution of expression values for the four time points, with disease status coloured as for the histograms. *Y* axis—expression (CPM, log_10_ scale). *X*-axis—time point. (**B**)* Coexpression of Class I MHC genes.* Histogram shows expression of *HLA-A* (red) and *HLA-B* (black) in each individual. *Y* axis—expression in CPM. *X* axis—individuals and time points. Each column represents one sample and the four time points for each individual are adjacent to each other and next to other family members. Upper bar: family (arranged as in Table S1). Lower bar—disease status (red: affected with Crohn’s disease or ulcerative colitis; black: unaffected sibling of an affected individual; blue: healthy donor with no family history of IBD). Some individuals express one or other of the genes, others express both and some express neither. In several families, members show different patterns

In the cluster analysis, the class II MHC regulator *CIITA*, and chaperone, *CD74*, were not correlated with class II MHC gene expression. *CD74* was highly and relatively uniformly expressed and clustered only with *HLA-DPA1 *(**Cluster 870**). Stringently co-regulated HLA genes were pairs located adjacent to each other in the genome, *HLA-B/HLA-C, HLA-A/HLA-F* and *HLA-DRB1/HLA-DRB5*, correlated with each other because of extreme variation between individuals. The expression of these gene pairs across the whole data set is shown Fig. 6A**,** sorted by family and separately by disease status. In each case, there are siblings with > 100-fold differences in expression, with examples of concordant and discordant sib-pairs that are not related to disease status. However, in overview, 38 of the 56 tested individuals over-expressed at least one of these HLA gene pairs in MDM. Figure 6B shows the levels of expression of *HLA-A* and *HLA-B*, where individuals may have similar or very different expression levels. Table 2 relates the HLA genotypes of each individual to the level of expression of four of these HLA genes. Expression of each of the correlated pairs was not strictly related to HLA genotype across the cohort but was correlated with HLA genotype in sib-pairs.

## Discussion

### Gene-specific variation in transcriptional regulation in monocyte-derived macrophages

The unifying concept that dysregulated monocyte–macrophage differentiation is an essential feature of intestinal inflammation has been widely-accepted (Reviewed in Hegarty et al. 2023). This project was based upon the concept that the differentiation of monocytes in response to CSF1 and a regulated response to microbial stimulus is a key event in adaptation to the pro-inflammatory environment of the lower GI tract. Based upon substantial enrichment identified in large-scale GWAS studies for IBD association with SNVs in the vicinity of the promoters of regulated macrophage-expressed genes (Baillie et al. 2017), we hypothesized that if they exist, regulatory variants affecting basal or inducible gene expression in monocytes differentiated in CSF1 would be prevalent in IBD families. In our data, the overall patterns of gene expression in stimulated macrophages clustered based upon time, reflecting the major changes induced by LPS, but there was no relationship with disease status or PRS (Figs. 1, S1, Tables S1C–F). Hence, the data provide no support for the recent proposal that IBD arises from a global hyper-responsiveness to TLR4 signals that distinguishes affected and unaffected individuals (Papoutsopoulou et al. 2019). Rather, consistent with the rationale for our approach, we identified very large differences (> 100-fold) between individuals in the basal or induced expression of specific genes. The magnitude of the variation detected in MDM is much greater than the 2–4-fold variation associated with SNV genotype in published eQTL for monocytes (Fairfax et al. 2014; Kim-Hellmuth et al. 2017; Richard et al. 2018). The large majority of transcripts detected in MDM in our data also varied within a 2–4-fold range (Tables S1C–F) and are likely associated significantly with *cis-*eQTL of small effect that would be detectable with a larger cohort. Indeed, if we apply the less stringent false discovery rate of < 0.05 as in the published study of blood monocytes (Fairfax et al. 2012) around 20% of probes associated with an eQTL in our data. Furthermore, the extreme variation between individuals was gene/locus-specific but with some exceptions was not detected in eQTL analysis (Table S3) and was not associated with local SNV genotype (Fig. S2) within the limits of detection in the current cohort. The variation we detected in MDM in the expression of the *CSF1R* gene, which is also high-expressed by microglia in the brain, may be related to the variable penetrance of heterozygous mutations linked to dominant leukoencephalopathy (Chitu et al. 2022). Amongst the hypervariable cytokine transcripts, *TNF*, a target for multiple therapeutic interventions, is an important exception. Aside from one individual with a delayed response, peak TNF expression at 2 h varied < three-fold between individuals (Table S1D). The lack of detectable SNV association does not exclude the possibility that at least some of the extreme variation is due to less common *cis*-acting variants of large effect that are enriched in IBD families but are not sampled by the ImmunoArray or fall below the arbitrary MAF cutoff of 0.2 (Li and Leal 2008). However, even with whole genome sequences and a much larger cohort or large multigenerational families it would be difficult to attribute causation to any variant (Momozawa et al. 2018; Sazonovs et al. 2022; Somineni et al. 2021).

We cannot say conclusively that the extreme variation we observed is specific to individuals who are susceptible to IBD. Ideally, the study would be extended to a larger cohort of affected individuals with high positive PRS compared to controls with negative PRS and no family history of IBD. However, there are two comparable published studies of macrophages that suggest the variation is indeed related to IBD. Moreno-Moral et al. (2018) compared the responses of MDM from 57 patients with systemic sclerosis to a control group of 15. Alasoo et al*. *(2018) identified conditional eQTL in macrophages derived from induced pluripotent stem cells from 87 individuals. Neither study revealed inter-individual variation on the scale we have observed in IBD families.

### Regulation of interferon-responsive genes in MDM

Aside from multiple pro-inflammatory cytokines and chemokines and inducible feedback regulators highlighted by our analysis, many other hypervariable regulated transcripts expressed in MDM can form the basis for a hypothetical link to IBD susceptibility. For example, *IFITM1, IFITM2* and *IFITM3* contribute to endocytic targeting of incoming virus particles, restricting virus replication (Spence et al. 2019). Each of these transcripts was high-expressed by monocytes and down-regulated by CSF1, enabling entry of influenza virus, expression of viral proteins and induction of pro-inflammatory genes (Clohisey et al. 2020). *IFITM3* variants are associated with severe disease in influenza infection and an *Ifitm3* knockout mouse is susceptible to low pathogenicity infections (Everitt et al. 2012). Mice lacking the entire IFITM locus develop spontaneous colitis (Alteber et al. 2018). In unstimulated MDM, *IFITM1* was barely detectable but there was residual expression of *IFITM2* and *IFITM3.* Each *IFITM* transcript was induced by LPS with differences in time course and magnitude, and large variations between individuals, but they were sufficiently correlated to form part of the interferon-response **Cluster 3,** correlated with variation in the rapid induction of *IFNB1* (Table S4) in the current data set. We also detected an eQTL affecting expression of the transcription factor *IRF5* (Table S3)*,* which could contribute to variable expression of target genes. Differential *IRF5* expression may be a consequence of a functional promoter variant. A relatively common 5 bp indel in the *IRF5* promoter was associated with increased IBD risk in a large Belgian cohort (Dideberg et al. 2007). Could the extreme variation in expression of these and other LPS-inducible interferon-responsive genes (including *CXCL9/10/11, IDO1, IFIT2, ISG15, ACOD1, RSAD2, OSAL, IFIH, ISG20*) predispose to hyper- or hypo-responsiveness to enteric viruses (Adiliaghdam et al. 2022) as the initial environmental trigger of chronic inflammation?

### Identification of MDM as potential antigen-presenting cells in acquired immune response

Subsets of macrophages in the human colonic lamina propria express class II MHC antigens and have high antigen presenting cell (APC) activity (Domanska et al. 2022). In the current study we have also identified novel inducible genes in CSF1-stimulated MDM potentially related to APC function and therefore relevant to dysregulated acquired T cell-mediated immune response in IBD (Clough et al. 2020; Graham and Xavier 2020; Martin et al. 2019). As discussed above, the ERAP1/ERAP2/LNPEP aminopeptidases are involved in antigen processing and variation is linked to multiple autoimmune diseases (Lopez de Castro 2018). The profound induction by LPS of transcripts encoding the chemokine receptor, CCR7, and its major ligand, CCL19, in MDM has not been reported previously. At each of the time points, *CCR7* expression varied between individuals (Table S1C–F). The literature on CCL19/CCR7 biology in IBD focuses exclusively on its expression by cells annotated as dendritic cells (DC) and the function in guiding these cells to draining lymph nodes (Alrumaihi 2022; Martin et al. 2019). Transcripts encoding inducible co-stimulatory/inhibitory molecules detected on active human APC (Hubo et al. 2013), CD83, CD80, CD40 and CD274 (PDL-1) were also inducible > 20 fold by LPS in MDM and highly variable between individuals (Table S1C–F). The macrophages of the lamina propria of mice, conflated with DC because of their expression of CD11c (*Itgax*) (see Summers et al. 2020) express CCR7 and migrate in response to CCL21 (Jang et al. 2006). As expected, the human monocytes differentiated in CSF1 expressed *ITGAX* (CD11c) and *ITGAM* (CD11b) mRNA. They also express *ITGAE* (CD103), commonly used as a marker of mouse and rat DC (Summers et al. 2020), but lack detectable *FLT3*, the receptor required for classical DC development. Studies in the rat have demonstrated that migrating “dendritic cells” in lymphatics draining the gut are derived from blood monocytes (Yrlid et al. 2006). We conclude that monocytes differentiated in CSF1 can be primed to respond to microbial agonists by becoming migratory APC.

### The association between HLA genotype and expression of specific transcripts

Of course, the primary determinant of APC activity is the expression of MHC molecules to enable presentation of antigenic peptides to T cell receptors. If monocyte differentiation in CSF1 provides a model for adaptation to the gut mucosa (Baillie et al. 2017), then the differential expression of both Class I and Class II MHC genes, independent of their antigen binding specificity, could contribute to a selective adaptive immune response to specific classes of mucosal antigens leading to inflammation. As noted in the introduction, shared haplotypes in the HLA region provide the greatest prediction of shared risk of IBD amongst members of the same family (Ahmad et al. 2006; Satsangi et al. 1996). This association is evident in the PRS data generated herein (Fig. S1). High-density SNV typing of the HLA region in > 32,000 individuals with IBD implicated several HLA alleles, with the greatest effect size associated with HLA-DRB1*01:03 in both CD and ulcerative colitis (Goyette et al. 2015). Seven of the individuals in our cohort (five affected, two unaffected) have this allele, all as heterozygotes. This includes three siblings in one family (MN11), with one unaffected individual. The discussion of these specific HLA associations with IBD susceptibility (Goyette et al. 2015) has focused on variation in the antigen-binding sites, with the presumption that these variants predispose in some way to response to a defined environmental trigger. There is also evidence of associations of class I MHC (specifically HLA-C) alleles with IBD (Jung et al. 2016) and of regulatory polymorphism affecting HLA-C expression (Vince et al. 2016). High HLA-C expression on peripheral blood leukocytes was associated with increased risk of CD (Apps et al. 2013). 11/56 individuals in our cohort expressed high levels of HLA-C (> 400 CPM) in MDM (compared to an average of < 2 CPM in non-expressors) but there was no correlation with disease status (Fig. 6). The relationships between HLA alleles and expression in relevant cell types has not been explored in the context of IBD. Raj et al. (2016) reported the existence of numerous regulatory polymorphisms affecting expression of Class II MHC genes in monocytes cultured in CSF2 (GM-CSF) (so-called monocyte-derived DC, moDC), as related to susceptibility to systemic lupus erythematosus. However, the effect sizes were small. By contrast, our analysis of MDM grown in CSF1 revealed apparent null expression of specific class I (*HLA-A, HLA-B, HLA-C, HLA-E, HLA-F*) and class II (*HLA-DRA, HLA-DRB1, HLA-DRB5, HLA-DQA1, HLA-DQB1, HLA-DMB, HLA-DPA1*) genes in certain individuals, linked to HLA haplotypes (Table 2, Fig. 6). HLA-C is of particular interest as the major determinant of natural killer (NK) cell recognition (Papuchova et al. 2019; Parham 2008). The small number of individual monocytes and MDM profiles in the FANTOM5 project also show > ten-fold variation in expression of each of these genes amongst individuals. These large differences could regulate acquired immune responses to microflora that might in turn initiate a chronic inflammatory state.

**Table 2:** HLA genotypes and level of expression of HLA genes

Individual ID	Status	HLA-A allele1	HLA-A allele2	HLA-A expression	HLA-B allele1	HLA-B allele2	HLA-B expression	HLA-C allele1	HLA-C allele2	HLA-C expression	HLA-DRB1 allele1	HLA-DRB1 allele2	DRB1 Expression
MN1AF	AF	A*01:01	A*02:01	0	B*08:01	B*40:01	++	C*03:04	C*07:01	0	DRB1*15:01	DRB1*13:02	+++
MN1UN	UN	A*01:01	A*68:01	0	B*08:01	B*44:02	0	C*07:04	C*07:04	+	DRB1*15:01	DRB1*11:01	++
MN5AF	AF	A*26:01	A*02:01	0	B*13:02	B*52:01	0	C*06:02	C*12:02	0	DRB1*15:02	DRB1*07:01	++
MN5UN	UN	A*02:03	A*01:01	0	B*13:02	B*38:02	0	C*06:02	C*07:02	+++	DRB1*16:02	DRB1*07:01	++
MN6AF	AF	A*02:05	A*02:05	0	B*50:01	B*15:01	+	C*18:01	C*06:02	0	DRB1*13:01	DRB1*07:01	0
MN6UN	UN	A*24:02	A*02:01	0	B*15:01	B*15:01	0	C*04:01	C*03:03	0	DRB1*13:01	DRB1*03:17	0
MN8AF	AF	A*25:01	A*11:01	+	B*39:01	B*35:01	+	C*04:01	C*12:03	0	DRB1*01:03	DRB1*07:01	+
MN8UN	UN	A*29:02	A*02:01	0	B*44:02	B*44:02	0	C*05:01	C*16:01	0	DRB1*04:01	DRB1*07:01	0
MN9AF	AF	A*03:01	A*29:02	+++	B*44:03	B*15:01	0	C*03:04	C*16:01	0	DRB1*15:01	DRB1*07:01	+++
MN9UN	UN	A*03:01	A*29:02	+++	B*44:02	B*40:01	0	C*05:01	C*16:01	0	DRB1*01:03	DRB1*07:01	+
MN11AFC	AF	A*31:01	A*02:01	0	B*39:01	B*07:02	+++	C*12:03	C*07:02	+++	DRB1*01:03	DRB1*04:04	+
MN11AFU	AF	A*31:01	A*02:01	0	B*39:01	B*07:02	+++	C*12:03	C*07:02	+++	DRB1*01:03	DRB1*04:04	+
MN11UN	UN	A*31:01	A*02:01	0	B*39:01	B*07:02	+++	C*12:03	C*07:02	+++	DRB1*01:03	DRB1*04:04	+
MN12AF	AF	A*01:01	A*01:01	0	B*08:01	B*08:01	0	C*07:01	C*07:01	0	DRB1*03:01	DRB1*03:01	0
MN12UN	UN	A*01:01	A*01:01	0	B*08:01	B*08:01	0	C*07:01	C*07:01	0	DRB1*03:01	DRB1*03:01	0
MN13AF	AF	A*03:01	A*02:01	+++	B*44:02	B*07:02	+++	C*07:02	C*05:01	+++	DRB1*01:03	DRB1*04:01	++
MN13UN	UN	A*11:01	A*11:01	++	B*44:02	B*35:01	0	C*04:01	C*05:01	0	DRB1*04:08	DRB1*14:103	0
MN14AF	AF	A*01:01	A*01:01	0	B*08:01	B*08:01	0	C*07:01	C*07:01	0	DRB1*03:01	DRB1*03:01	0
MN14UN	UN	A*01:01	A*03:01	+++	B*08:01	B*35:03	0	C*07:01	C*04:01	0	DRB1*03:01	DRB1*10:01	0
MN15AF	AF	A*03:01	A*02:01	+++	B*18:01	B*57:01	0	C*06:02	C*07:01	0	DRB1*11:04	DRB1*07:01	0
MN15UN	UN	A*32:01	A*02:01	0	B*44:02	B*15:01	0	C*05:01	C*03:03	0	DRB1*12:01	DRB1*04:01	0
MN16AF	AF	A*30:02	A*03:01	+++	B*18:01	B*53:01	0	C*04:01	C*05:01	0	DRB1*11:01	DRB1*03:17	0
MN16UN	UN	A*30:02	A*03:01	+++	B*18:01	B*53:01	0	C*04:01	C*05:01	0	DRB1*11:01	DRB1*03:17	0
MN17AF	AF	A*02:01	A*03:01	0	B*44:02	B*15:01	0	C*05:01	C*03:03	0	DRB1*01:03	DRB1*04:01	++
MN17UN1	UN	A*01:01	A*01:01	0	B*08:01	B*52:01	0	C*07:01	C*12:02	0	DRB1*03:01	DRB1*15:02	+++
MN17UN2	UN	A*01:01	A*01:01	0	B*08:01	B*52:01	0	C*07:01	C*12:02	0	DRB1*03:01	DRB1*15:02	+++
MN18AFC	AF	A*02:01	A*01:01	0	B*57:01	B*35:01	0	C*06:02	C*18:01	0	DRB1*11:01	DRB1*07:01	0
MN18AFU	AF	A*02:01	A*31:01	0	B*57:01	B*40:01	++	C*06:02	C*03:04	0	DRB1*11:01	DRB1*07:01	0
MN19AF	AF	A*24:02	A*02:01	0	B*49:01	B*15:01	+	C*07:01	C*03:04	0	DRB1*11:02	DRB1*01:01	+
MN19UN	UN	A*25:01	A*02:01	0	B*18:01	B*15:01	0	C*12:03	C*03:04	0	DRB1*07:01	DRB1*01:01	+
MN20AF	AF	A*30:04	A*03:01	+++	B*51:08	B*35:01	0	C*04:01	C*16:02	0	DRB1*01:01	DRB1*13:02	+
MN20UN	UN	A*31:01	A*02:01	0	B*51:01	B*44:02	0	C*15:02	C*16:01	0	DRB1*04:07	DRB1*07:01	0
MN21AF	AF	A*68:01	A*02:01	0	B*51:01	B*40:01	++	C*16:02	C*03:04	0	DRB1*08:01	DRB1*04:07	0
MN21UNa	UN	A*68:01	A*02:01	0	B*51:01	B*40:01	++	C*16:02	C*03:04	0	DRB1*08:01	DRB1*04:07	0
MN21UNb	UN	A*68:01	A*02:01	0	B*51:01	B*40:01	++	C*16:02	C*03:04	0	DRB1*08:01	DRB1*04:07	0
MN22AF	AF	A*01:01	A*03:01	+++	B*37:01	B*07:02	+++	C*07:02	C*06:02	+++	DRB1*15:01	DRB1*14:01	++
MN22UN	UN	A*01:01	A*11:01	+	B*08:01	B*35:01	0	C*07:01	C*04:01	0	DRB1*03:01	DRB1*14:01	0
MN23AF	AF	A*24:02	A*02:01	0	B*44:02	B*18:01	0	C*16:04	C*07:01	0	DRB1*11:04	DRB1*13:01	0
MN23UNa	UN	A*24:02	A*02:01	0	B*44:02	B*18:01	0	C*16:04	C*07:01	0	DRB1*11:04	DRB1*13:01	0
MN23UNb	UN	A*24:02	A*24:02	0	B*44:02	B*51:01	0	C*16:04	C*01:02	0	DRB1*11:04	DRB1*03:01	0
MN24AF	AF	A*24:02	A*03:01	+++	B*57:01	B*07:02	+++	C*06:02	C*07:02	+++	DRB1*15:01	DRB1*07:01	+++
MN24UN	UN	A*32:01	A*02:01	0	B*38:01	B*40:02	+	C*02:02	C*12:03	0	DRB1*10:01	DRB1*07:01	0
MN25AF1	AF	A*03:01	A*03:01	+++	B*07:02	B*47:01	+++	C*07:02	C*06:02	+++	DRB1*15:01	DRB1*04:05	+++
MN25AF2	AF	A*11:01	A*03:01	+++	B*18:01	B*47:01	0	C*07:01	C*06:02	0	DRB1*15:01	DRB1*04:05	+++
MN25UN	UN	A*03:01	A*03:01	+++	B*07:02	B*35:01	++	C*07:02	C*04:01	++	DRB1*15:01	DRB1*01:01	+++
MN26AF	AF	A*01:01	A*02:01	0	B*44:02	B*57:01	0	C*06:02	C*05:01	0	DRB1*04:01	DRB1*07:01	0
MN26UN	UN	A*30:02	A*31:01	+	B*35:01	B*18:01	0	C*04:01	C*05:01	0	DRB1*04:07	DRB1*03:01	0
MN27AF	AF	A*03:01	A*24:02	+++	B*08:01	B*47:01	0	C*06:02	C*07:06	0	DRB1*03:01	DRB1*03:01	0
MN27UN1	UN	A*03:01	A*24:02	++	B*08:01	B*47:01	0	C*06:02	C*07:06	0	DRB1*03:01	DRB1*03:01	0
MN27UN2	UN	A*03:01	A*24:02	++	B*08:01	B*47:01	0	C*06:02	C*07:01	0	DRB1*03:01	DRB1*03:01	0
MN2HD	HD	A*01:02	A*02:01	0	B*08:01	B*44:02	0	C*05:01	C*07:01	0	DRB1*15:01	DRB1*03:01	+++
MN5HD	HD	A*03:01	A*24:02	++	B*38:01	B*35:03	0	C*04:01	C*12:03	0	DRB1*15:02	DRB1*13:01	++
MN7HD	HD	A*68:01	A*24:02	0	B*51:01	B*07:02	+++	C*14:02	C*07:02	+++	DRB1*15:01	DRB1*10:01	+++
MN8HD	HD	A*03:01	A*02:01	++	B*08:01	B*44:02	0	C*05:01	C*07:01	0	DRB1*04:01	DRB1*07:01	0
MN9HDa	HD	A*32:01	A*02:05	0	B*35:03	B*57:01	0	C*18:02	C*06:02	0	DRB1*11:01	DRB1*07:01	0
MN9HDb	HD	A*32:01	A*02:01	0	B*35:03	B*08:01	0	C*04:01	C*07:01	0	DRB1*11:01	DRB1*03:01	0

HLA genotypes were derived from RNA2HLA (see “Materials and methods”). Expression levels are given as indicated for each gene

*HLA-A*: < 250 CPM = 0; 250 CPM ≤ x < 1000 CPM =+; 1000 CPM ≤ x < 3000 CPM =++; ≥ 3000 CPM =+ ++. The maximum value was 8227 CPM and the time point for maximum expression was 21 h

*HLA-B*: < 250 CPM = 0; 250 CPM ≤ x < 1000 CPM =+; 1000 CPM ≤ x < 3000 CPM =++; ≥ 3000 CPM =+ ++. The maximum value was 6948 CPM and the time point for maximum expression was 21 h

*HLA-C*: < 100 CPM = 0; 100 CPM ≤ x < 500 CPM =+; 500 CPM ≤ x < 1500 CPM =++; ≥ 1500 CPM =+ ++. The maximum value was 3132 CPM and the time point for maximum expression was 21 h

*HLA-DRB1*: < 25 CPM = 0; 25 CPM ≤ x < 100 CPM = +; 100 CPM ≤ x < 300 CPM =++ ≥ 300 CPM =+ ++. The maximum value was 704 CPM and the time point for maximum expression was 0 h for most individuals

AF, affected with Crohn’s disease or ulcerative colitis; UN, unaffected sibling of an affected individual; HD, healthy donor with no family history of IBD

## Conclusion

This study has not identified a common global pattern of dysregulated variation in macrophage activation that distinguishes affected and unaffected individuals or discordant sib-pairs from each other. What it has done is to confirm that in macrophages differentiated in CSF1 there are very large inter-individual differences in basal and LPS-inducible expression of specific pro-inflammatory or anti-inflammatory genes. Some of the variation may be due to less common *cis*-acting variants of large effect that are not captured by SNP genotyping (Li and Leal 2008) and some may be a downstream consequence of genetic variation in expression of key regulators such as IL1B and IFNB1 (Table S4). However, given the complex transcriptional network and feedback regulation involved in macrophage differentiation and activation, exemplified by the transcriptional network analysis in the current study (Fig. 1) the expression of each transcript may be a complex trait, as inferred in analysis of many other cellular systems (Albert et al. 2018; Albert and Kruglyak 2015; Flynn et al. 2022). That alternative is consistent with the unifying concept that the heritability of complex traits derives from numerous weak *trans* effects that converge on a set of trait-relevant target genes (Vosa et al. 2021). The substantial variation we have observed could potentially represent an intermediate phenotype underlying heritable susceptibility to chronic inflammation in response to an environmental trigger as well as the clinical heterogeneity of IBD (Sudhakar et al. 2021). If that is the case, with a sufficiently large cohort, it might be possible to define PRS linked to the expression of individual pro-inflammatory genes (e.g. *IFNB1*, *IL1B, IL6, TNF, CXCL1* etc.) in MDM that could have a clinical predictive value. The concept that each family is unique (i.e. common disease, rare variant (Li and Leal 2008)) has been termed the Anna Karenina hypothesis for the genetics of complex disease (McClellan and King 2010). The corollary of our finding that each individual has a unique macrophage transcriptome is that IBD may, in fact, be many different diseases with a diverse underlying molecular basis contributing to the clinical heterogeneity and variation in treatment efficacy (Verstockt et al. 2022) and to the nature of the environmental trigger.

### Supplementary Information

Below is the link to the electronic supplementary material.

Supplementary file1 (DOCX 1966 kb)

Supplementary file2 (XLSX 42883 kb)

Supplementary file3 (XLSX 16 kb)

Supplementary file4 (XLSX 21442 kb)

Supplementary file5 (XLSX 21217 kb)

Supplementary file6 (XLSX 21339 kb)

Supplementary file7 (XLSX 21225 kb)

Supplementary file8 (XLSX 5611 kb)

Supplementary file9 (XLSX 827 kb)

Supplementary file10 (XLSX 127 kb)

## Data Availability

All of the processed RNA-seq data are presented in Table S1. The raw RNA-seq sequence files that support the findings of this study are available on reasonable request from the corresponding authors. The data are not publicly available due to privacy and ethical restrictions.
