# Supplementary material for: The relationship between extreme inter-individual variation in macrophage gene expression and genetic susceptibility to inflammatory bowel disease
Source: Hum Genet. 2024 Feb 29;143(3):233–61. doi: 10.1007/s00439-024-02642-9 (PMC11043138; doi:10.1007/s00439-024-02642-9)

**Supplementary Figures**

**Figure S1. Genotype and normalised polygenic risk scores (PRS) for individuals in the study.**

Principal components analysis (PCA) plot was generated using ggplot2 package in the R statistical environment (see Materials and Methods) with a minor allele frequency threshold of > 0.01. Polygenic risk scores were calculated as described in Materials and Methods. **Figure S1A** plots the first two components of the PCA. The ID of three outlier families is given. **Figure S1B** shows risk scores for the complete SNV sets. See also **Table S1A** for risk score values for all individuals. **Figure S1C** shows the risk scores for SNV sets after excluding SNV located within the MHC region. Y axis shows the mean risk score; error bars represent the 95% confidence interval (CI) for each estimate. The X axis displays the GWAS p-value threshold used to select qualified SNPs for PRS calculation. Red: individuals affected with Crohn’s disease or ulcerative colitis; black: unaffected siblings of an affected individual and healthy donors with no family history of IBD.

**
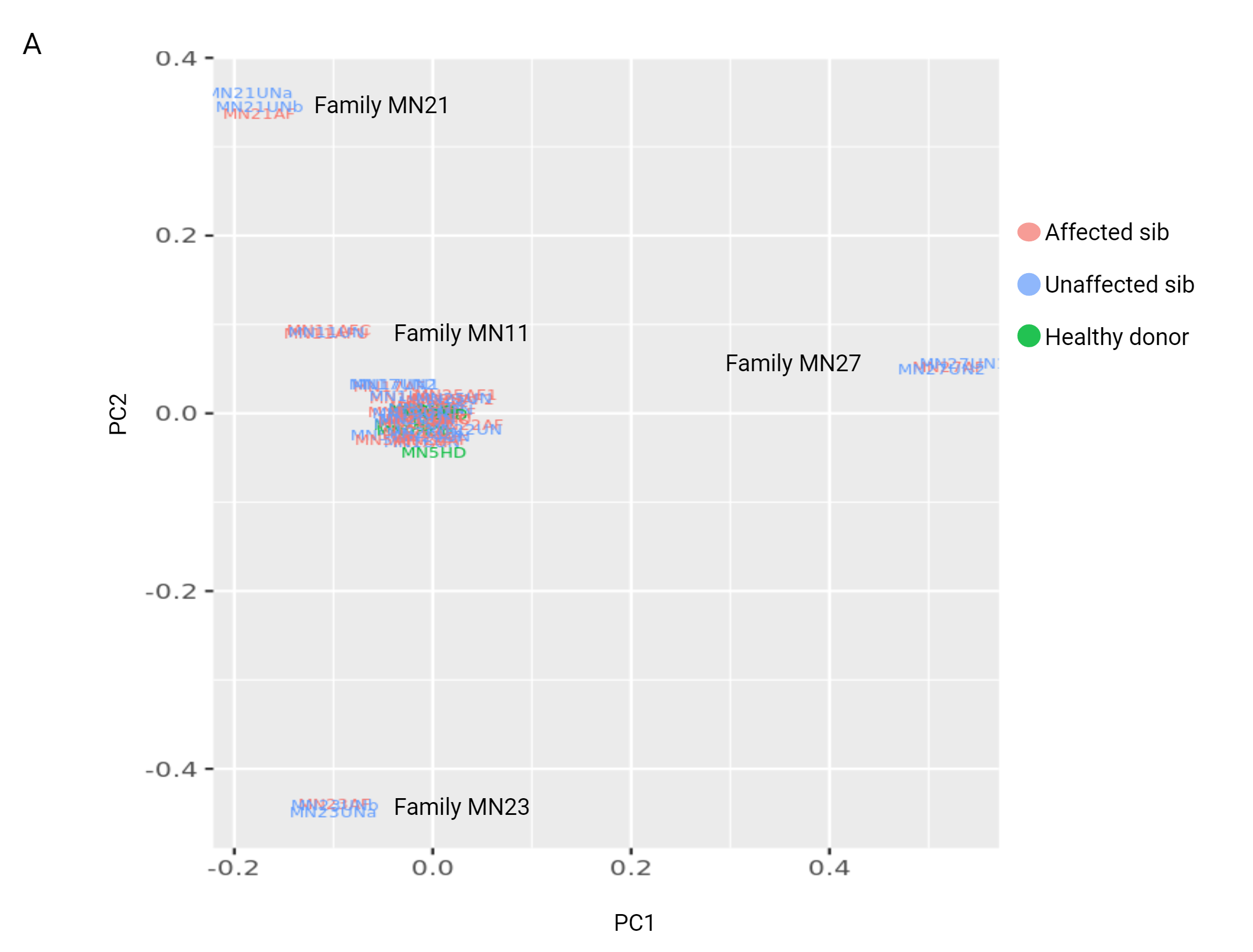
**

**
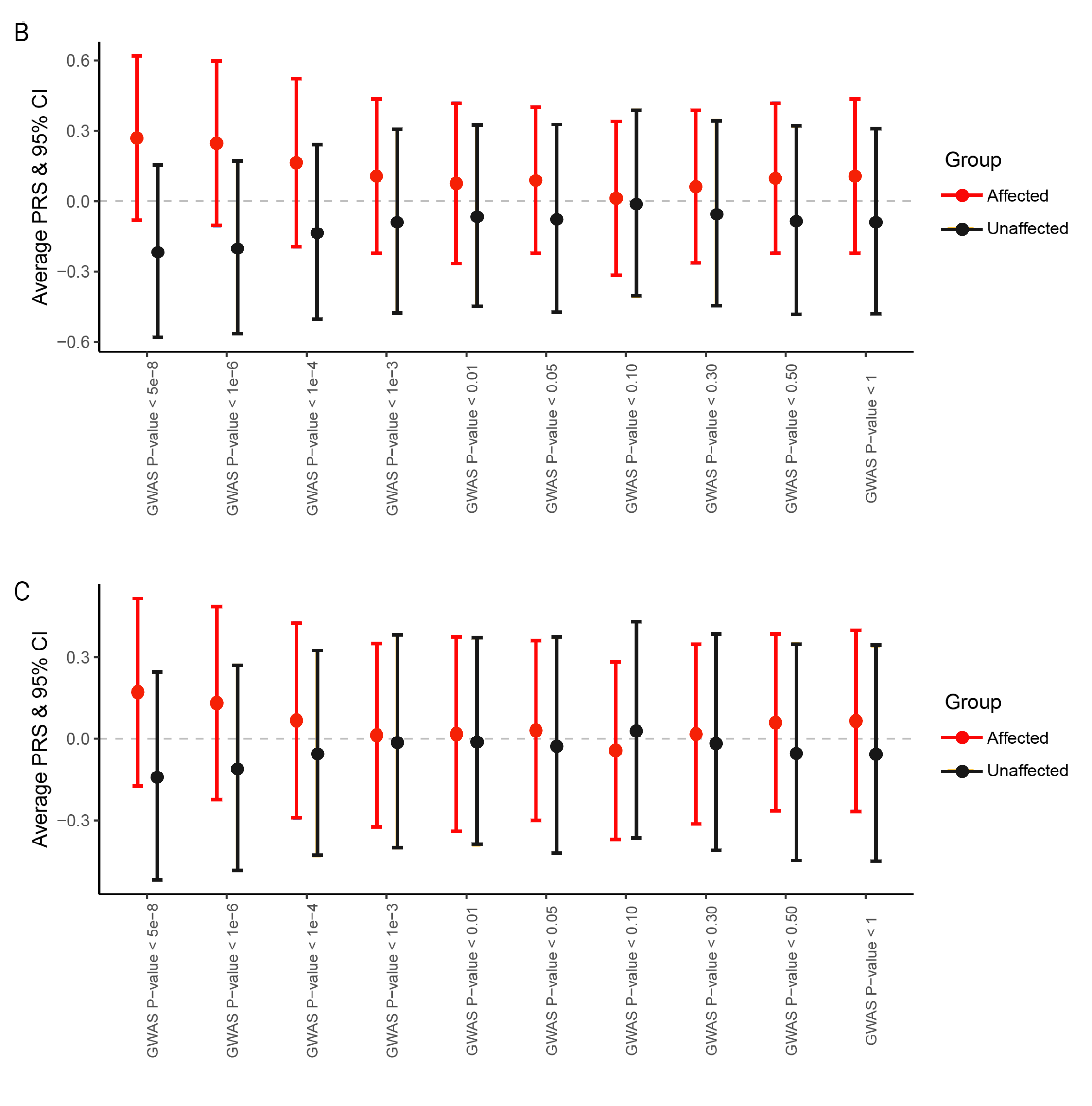
**

**Figure S2. SNV associated with expression of selected genes.**

In all figures, the Y axis shows the expression level (CPM) and the X axis shows the three genotypes for the SNV. Each dot represents one sample (red: affected with Crohn’s disease or ulcerative colitis; black: unaffected sibling of an affected individual; blue: healthy donor with no family history of IBD).

*Figure S2A.* Expression of *IL6* SNV rs1474347 across the time course. There was no association between genotype and expression at any time point (Kruskal-Wallis test). There were several other SNV in apparent linkage disequilibrium with rs1474347 which gave the same result. *IL6* rs2069824, which was not in disequilibrium with these SNV, also showed no association between genotype and expression at any time point (Kruskal-Wallis test).

*Figure S2B.* Expression of *IL10* SNV rs1518111 across the time course. There was no association between genotype and expression at any time point (Kruskal-Wallis test). rs1800871 was in linkage disequilibrium with this SNV and gave the same result.

*Figure S2C.* Expression of *IRF2* SNV rs793798 across the time course. There was no association between genotype and expression at any time point (Kruskal-Wallis test). *IRF2* rs6812407, which was not in disequilibrium with this SNV, also showed no association between genotype and expression at any time point (Kruskal-Wallis test).

*Figure S2D.* Expression of *IRF7* SNV imm_11_609789 across the time course. Kruskal-Wallis test showed significant association between genotype and expression at 21 hrs (p-value = 0.0016) and marginally significant association at 7 hrs (p-value = 0.0135). Because there were very few homozygotes for the rare allele (A), we also analysed the association of the more common genotypes (CC and AC) with expression, by Mann-Whitney U test. This showed significant associations at 0, 7 and 21 hrs (p-value = 0.0184, 0.0024 and 0.0002 respectively) though not at 2 hrs (p-value = 0.0942).


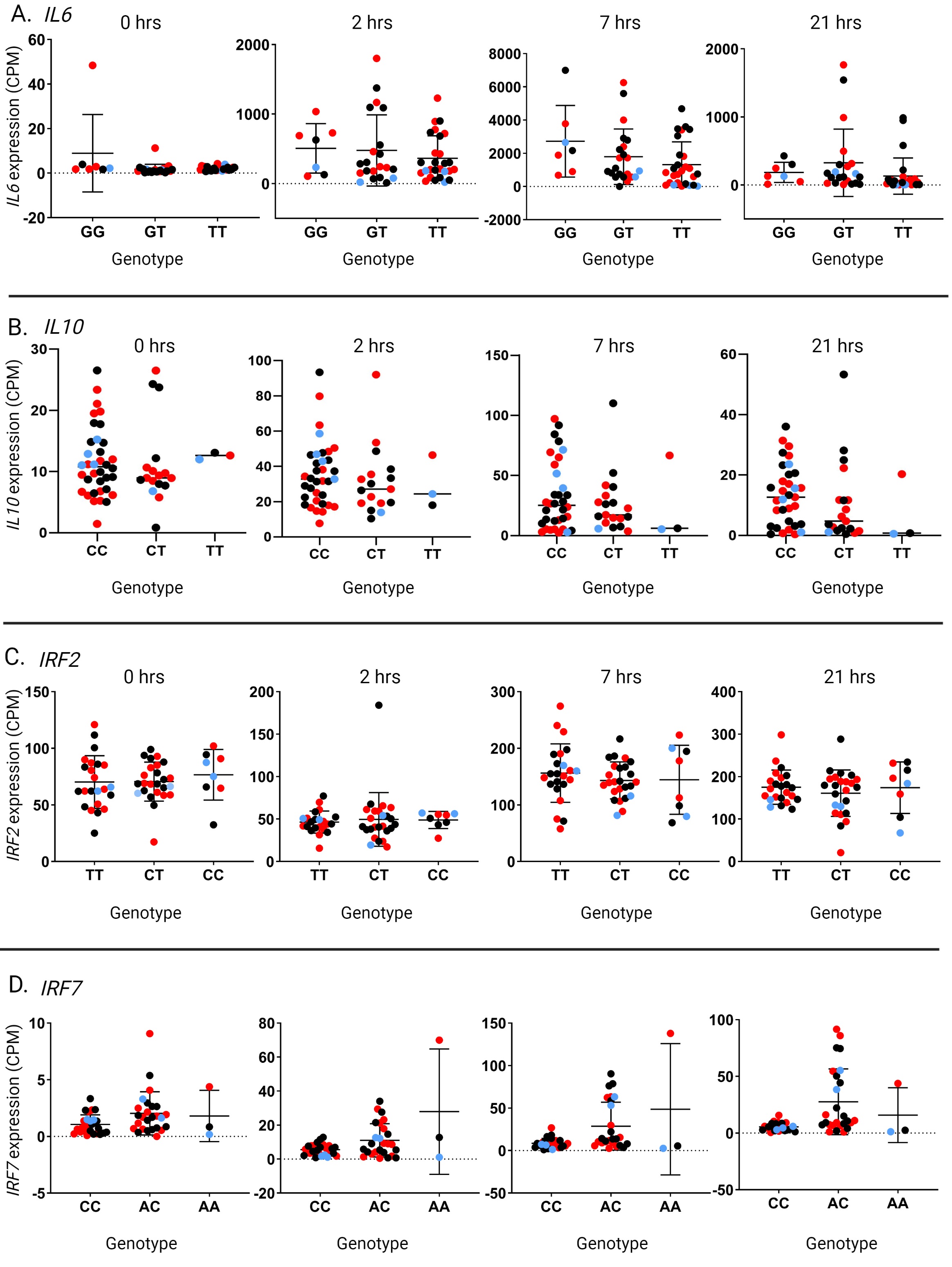


**Figure S3. Expression of chemokine genes.**

*Figure S3A.* Expression of *CXCL1* SNV rs2886927 across the time course. Y axis shows the expression level (CPM) and the X axis shows the three genotypes. Each dot represents one sample (red: affected with Crohn’s disease or ulcerative colitis; black: unaffected sibling of an affected individual; blue: healthy donor with no family history of IBD). There was no association between genotype and expression at any time point (Kruskal-Wallis test). SNV rs3117604 which was in linkage disequilibrium (one individual had an inconsistent genotype) also showed no significant association between expression and genotype.

*Figure S3B.* Correlation matrix for expression of chemokine genes. Numbers show the Spearman correlation coefficients for pairs of genes.


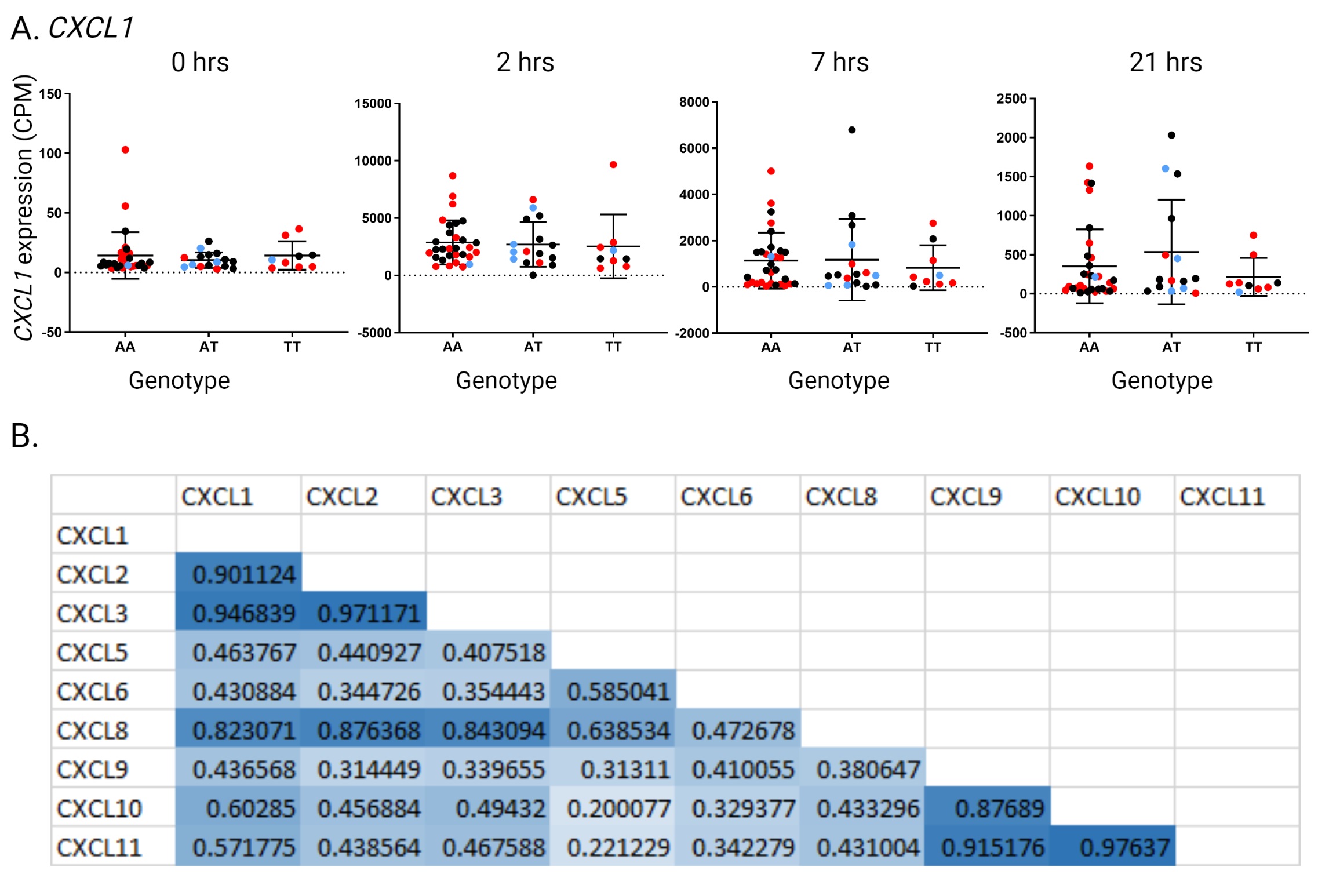

Supplement: Supplementary file 1 — Supplementary file1 (DOCX 1966 kb) [file 439_2024_2642_MOESM1_ESM.docx]
